# Retrotransposons as Drivers of Mammalian Brain Evolution

**DOI:** 10.3390/life11050376

**Published:** 2021-04-22

**Authors:** Roberto Ferrari, Nicole Grandi, Enzo Tramontano, Giorgio Dieci

**Affiliations:** 1 Department of Chemistry, Life Sciences and Environmental Sustainability, University of Parma, 43124 Parma, Italy; roberto.ferrari1@unipr.it; 2 Laboratory of Molecular Virology, Department of Life and Environmental Sciences, University of Cagliari, Cittadella Universitaria di Monserrato, 09042 Monserrato, Italy; nicole.grandi@unica.it (N.G.); tramon@unica.it (E.T.); 3 Istituto di Ricerca Genetica e Biomedica, Consiglio Nazionale delle Ricerche, 09042 Monserrato, Italy

**Keywords:** SINE, ERV, LINE-1, transposable elements, enhancer, exaptation, neocortex, noncoding RNA, neuron

## Abstract

Retrotransposons, a large and diverse class of transposable elements that are still active in humans, represent a remarkable force of genomic innovation underlying mammalian evolution. Among the features distinguishing mammals from all other vertebrates, the presence of a neocortex with a peculiar neuronal organization, composition and connectivity is perhaps the one that, by affecting the cognitive abilities of mammals, contributed mostly to their evolutionary success. Among mammals, hominids and especially humans display an extraordinarily expanded cortical volume, an enrichment of the repertoire of neural cell types and more elaborate patterns of neuronal connectivity. Retrotransposon-derived sequences have recently been implicated in multiple layers of gene regulation in the brain, from transcriptional and post-transcriptional control to both local and large-scale three-dimensional chromatin organization. Accordingly, an increasing variety of neurodevelopmental and neurodegenerative conditions are being recognized to be associated with retrotransposon dysregulation. We review here a large body of recent studies lending support to the idea that retrotransposon-dependent evolutionary novelties were crucial for the emergence of mammalian, primate and human peculiarities of brain morphology and function.

## 1. Introduction

A large fraction of most eukaryotic genomes is constituted by transposable elements (TEs), interspersed repeats of which the high copy number reflects mobile DNA integration events that occurred countless times throughout evolutionary history [1]. Although they represent a constant challenge for genome stability, TEs have at the same time introduced potentially fruitful changes into genomes both by driving genomic rearrangements (resulting, for example, in gene duplication) and by exaptation of TE-derived sequences [2,3,4]. Since TEs replicate as genomic parasites, eukaryotic host organisms have co-evolved TE silencing systems largely based on the deposition of repressive epigenetic marks, of which the effect on the epigenome accompanied the effect of TE on the genome over the course of evolution [5]. By allowing TE retention, TE silencing mechanisms have provided genomes with large pools of latent functional elements poised for exaptation [6]. While the so-called DNA transposons employ a mechanism directly moving DNA segments from one genomic location to another, the heterogeneous class of TEs referred to as retrotransposons (or retroelements) do so through reverse transcription of an RNA copy of the original element, thereby affecting genome composition through the constant introduction of new DNA material. The peculiar ability of retrotransposon systems to support conversion from RNA to DNA incessantly contributes new sequences, potentially encoding new protein/RNA molecules or providing new *cis*-regulatory functions that selection can act on to produce genomic and organismal innovations [7,8].

The last 15 years have seen a remarkable spurt of studies exploring the idea that retrotransposon activity affects brain development and function in mammals, through the promotion of somatic mosaicism in the brain [9,10], the generation of novel transcripts and proteins playing diverse roles in neuron biology [11,12], as well as the seeding of *cis*-regulatory elements, affecting transcription factor-dependent gene regulation, and of boundary elements, participating in three-dimensional (3D) genome architecture [13,14]. The deep involvement of retrotransposons in brain biology also makes them a source of vulnerability and disease [15,16]. All these indications of the pervasive influence of retrotransposons on brain biology consolidate the idea that the evolution of the nervous system, of which the results include the uniquely evolved human brain, has retrotransposons among its driving factors [13,17,18].

In this review, we intend to provide a comprehensive, updated and reasoned picture of the impact of retrotransposons on brain evolution in mammals, on which most relevant studies have been focused. We start with brief and updated accounts the existing general knowledge about retrotransposons and their impact on genome evolution and about key genomic innovations underpinning brain evolution in mammals. We then move on to a detailed discussion of recent studies which are increasingly revealing the pervasive contribution of the different groups of retrotransposons to the evolution of distinguishing brain features in mammals and particularly in humans.

## 2. Retrotransposons and Their Impact on Mammalian Genome Evolution

### 2.1. Retrotransposons in Mammalian Genomes

TEs are repetitive DNA sequences, typically ranging in length from 100 to 10,000 bp, capable of colonizing new genomic locations with copies of themselves. Based on their mode of transposition, with or without an RNA intermediate, TEs are split into two major classes: the eukaryote-specific class I TEs, or retrotransposons, which mobilize via a “copy-and-paste” mechanism involving reverse transcription of an RNA copy of a source element, and the class II TEs or DNA transposons, mobilizing without reverse transcription mainly via a “cut-and-paste” mechanism [4]. The increase in TE copy number in genomes is primarily due to their capacity for vertical inheritance through the germline, a property giving TEs great potential for the generation of evolutionary novelties. Indeed, an enormous amount of studies since their discovery has mitigated the emphasis on TEs as a useless form of parasitic DNA, by revealing their irreplaceable contribution to genome structure, information content and regulation [19,20], thus substantially corroborating early, far-seeing hypotheses about their role [21,22] (see [23] for a historical perspective). The other side of the coin is that TE mobility has the potential to disrupt functional genetic elements in both germinal and somatic cells, thus leading to disease [24,25]. It is thus not surprising that retrotransposons coevolve with mechanisms counteracting retrotransposition [26,27,28].

**Figure 1:**
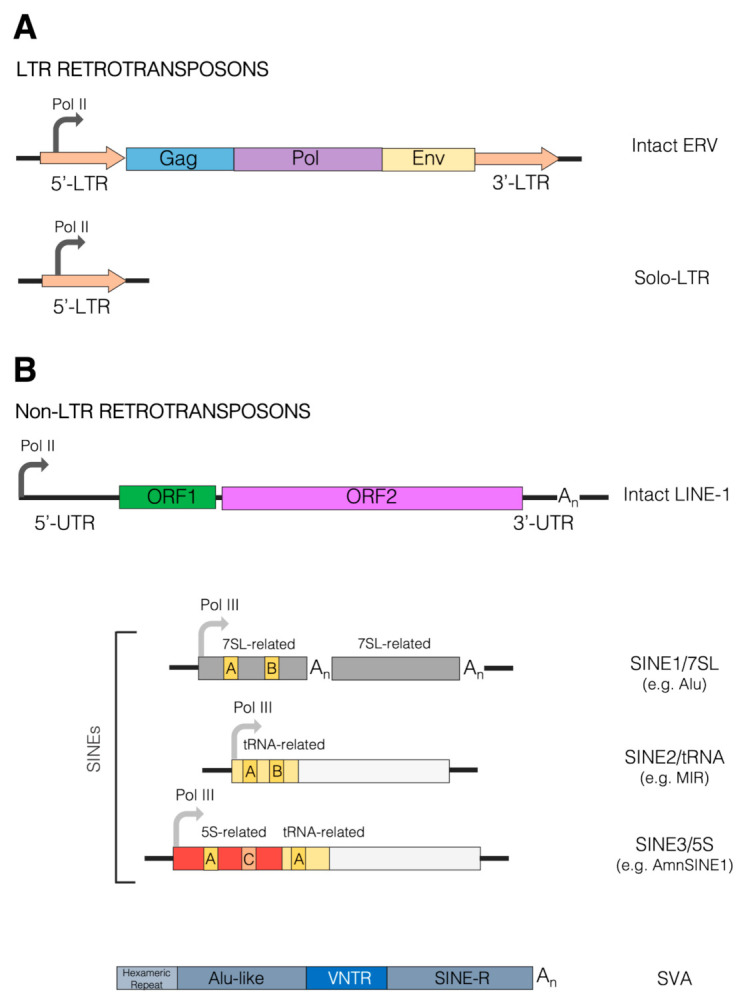
Schematic view of retrotransposons. Retrotransposons are divided into two main classes depending on the presence of long terminal repeat (LTR) regions. (**A**) LTR retrotransposons are also generally referred to as endogenous retroviruses (ERVs). Their full-length sequence (top) is schematically composed of a 5’-LTR (orange arrow) containing the RNA polymerase II (Pol II) promoter, from which the entire unit is transcribed. The coding region of the transcribed ERV spans three main genes, such as Gag, Pol and Env, represented by the blue, purple and yellow boxes, respectively. The coding region is followed by the 3’-LTR sequence (orange arrow); 5’ and 3’ LTRs are formed from viral RNA ends during reverse transcription and are identical at the time of integration. Intact LTR retrotransposons are autonomous, as they encode for the protein machinery required for their reverse transcription and integration. Many LTR retrotransposons are incomplete, however. In extreme cases (bottom), the recombination between 5’ and 3’ LTRs of the same provirus can reduce ERV sequences to a solitary LTR only, from which transcripts can originate by virtue of the Pol II promoter embedded within the LTR (orange arrow). (**B**) Non-LTR retrotransposons include both autonomous (LINE) and non-autonomous (SINE, SVA) classes, illustrated in the upper and lower parts of the panel, respectively. LINEs, predominantly represented by the LINE-1 (L1) group in humans, harbor a 5’-UTR, containing the Pol II promoter from which they are transcribed (curved arrow). The coding region of the transcribed LINE-1 is composed of two main open-reading frames, ORF1 and ORF2, coding for the homonymous proteins (green and pink boxes). The 3’-UTR of the LINE-1 contains a poly(A) tract (A_n_). SINEs are further divided into three main groups or clades: SINE1/7SL, SINE2/tRNA and SINE3/5S, depending on the type of ancestral sequence from which they originated, specifically the 7SL RNA, the tRNA and the 5S RNA sequence. Consistent with their origin, SINEs contain internal Pol III promoters. Alu elements, the most numerous SINEs in humans, belong to the SINE1/7SL group as they contain two 7SL-related moieties (gray boxes). The upstream 7SL-related moiety harbors A- and B-box internal control elements, recognized by the Pol III-specific transcription factor TFIIIC (yellow boxes) in their left 7SL-related moiety. The two moieties are also separated by an A-rich tract (A_n_). Another poly(A) tract is found at the end of the SINE. SINEs of the SINE2/tRNA group harbor A- and B-boxes in their tRNA-related upstream moiety (yellow boxes), followed by sequences of diverse origins. A noteworthy example of this group is represented by the mammalian-wide interspersed repeat (MIR) elements. SINE3/5S elements are exemplified in the Figure by AmnSINE1, formed by an upstream 5S-derived moiety (red box), containing the 5S-specific A- and C-box internal promoter elements, followed by a tRNA-related fragment (yellow). Represented in the bottom part of the panel is the structure of an SVA element, consisting of (from the 5’ to 3’ end) a hexameric repeat region, an Alu-related region, a variable number tandem repeat (VNTR) region, and a SINE-R sequence sharing homology with human endogenous retrovirus HERV-K10.

Each class of TEs comprises different clades/superfamilies and families, the diversity and complexity of which has prompted decades-long identification and classification efforts [29,30]. In particular, class I TEs are typically subdivided into long terminal repeat (LTR) and non-LTR retrotransposons, with the former displaying a close relationship with retroviruses and other reverse-transcribing viruses (Figure 1). TEs of both class I and II can further be classified as either autonomous or non-autonomous, the former having the ability to self-mobilize, the latter relying on the enzymatic machinery of other TEs for mobilization. Such a distinction is especially relevant in the case of non-LTR retrotransposons, which can be broadly divided into the autonomous elements referred to as long interspersed elements (LINEs) and the non-autonomous short interspersed elements (SINEs) [4]. SINEs are known to exploit the LINE retrotransposition machinery for mobilization, often facilitated by 3′ end sequence similarity between LINE and SINE partners [31,32]. Although LTR and LINE retrotransposons are transcribed by the RNA polymerase II machinery, assisted by a few sequence-specific transcription factors (TFs), most SINEs are transcribed by RNA polymerase III due to the presence of internal control regions (A box and B box) recognized by the Pol III-specific basal transcription factor TFIIIC [33,34]. Indeed, the evolutionary origin of most SINEs has been traced back to Pol III-transcribed genes coding for abundant small RNAs, such as tRNA, 5S rRNA and 7SL RNA, all employing TFIIIC as a sequence-specific DNA binding protein essential for transcription complex assembly [35]. Accordingly, SINEs are generally divided into SINE1/7SL, SINE2/tRNA and SINE3/5S (Figure 1), to which the more recently identified SINEU (derived from U1 and U2 snRNAs) have been added [29].

In the last decade, mainly due to high-throughput sequencing and TE annotation advancements, TEs have been disclosed as a major component of vertebrate genomes, strongly contributing to their diversity [36]. In mammals, which are the best-studied vertebrates in terms of TE biology, the mobilome (defined as the whole set of TEs in the genome) generally display distinguishing features among vertebrates. TEs account for more than 50% of the size of many mammalian genomes, with a preponderance of retrotransposons, which are present in extremely large copy numbers, a minimized content of DNA transposons and a low subfamily diversity compared to other vertebrates [36]. For example, in the two most intensively studied mammals, humans and mice, DNA transposons represent approximately 1.2–3% of the genome sequence, to be compared with the 40–45% of retrotransposons. Among the latter, LINEs contribute 20–22% of the genome sequence in both humans and mice, with LINE-1 (or L1) being the most abundant subfamily, contributing ~17% of the genome sequence. The relative abundance of SINEs and LTR retrotransposons differs markedly between the two mammals, however. In humans, LTR retrotransposons and SINEs represent ~8% and ~13% of the genome, respectively, whereas in mice the corresponding values are 12% and 8%, respectively [37,38].

The L1 family, perhaps the most evolutionarily successful retrotransposon family in mammals, has been a resident of their genomes since early in mammalian radiation, and is likely to have undergone recurrent cycles of adaptation and innovation, leading to the persistence of a single successful lineage [39]. In contrast, SINEs, whose expansion is free from the need to code for a retrotransposition machinery, did not expand continuously from a single, evolutionarily successful family. Instead, novel SINEs have arisen multiple times in the evolution of mammals and, more broadly, of vertebrates [36]. The diversity of lineage-specific SINE families that emerged during mammalian evolution is evident when looking at the distribution of SINE families across major mammal groups (see, for example, [13]). In particular, the most numerous human SINEs, represented by the SINE1/7SL Alu elements (~1.1 × 10^6^ copies), are primate-specific. A comparative study of mobile element insertions in human and great ape genomes revealed that, during recent human/great ape evolution, the most variable form of genetic variation is represented by Alu retrotransposition, with remarkable increases and decreases occurring over very short evolutionary times [40]. The recently discovered, composite SVA elements—evolutionarily young, SINE-derived retrotransposons which include subfamilies restricted to the human lineage—are also specific to primates [1,41,42]. In mice, the most numerous SINE family is represented by the 7SL-derived, monomeric B1 elements (~5.6 × 10^5^ copies), closely followed by the tRNA-derived B2 elements (~3.5 × 10^5^ copies) and by the B1-and tRNA-derived B4 elements (3.9 × 10^5^ copies) [37,43,44]. More generally, drastically different retrotransposon landscapes characterize the genomes of even closely related taxa, and speciation events and the expansion of new retrotransposon families are often correlated, all pointing to the mobilome as a driver of organism diversification [45]. This does not exclude the existence of conserved retrotransposon subfamilies that appeared at very early stages of mammalian evolution, such as the tRNA-derived SINEs referred to as mammalian-wide interspersed repeats (MIRs), which were actively propagating prior to the radiation of mammals and before placental mammals separated. Although they are retropositionally inactive, MIRs still represent the second most numerous SINE subfamily in humans [46,47,48].

Concerning LTR retrotransposons, as mentioned above, they derive from ancestral retroviral infections sustained by exogenous retroviruses that have now gone extinct, except for a few exceptional examples of ongoing endogenization [49]. Having originated from proviral integrations, these elements display a typical retroviral structure—presenting two LTRs that flank the three main genes *gag*, *pro-pol* and *env*—and are hence also named endogenous retroviruses (ERVs) (Figure 1). ERVs are present in all vertebrate genomes, constituting around 10% of the diverse species’ DNA, and have provided important contributions to their hosts over the course of evolution [50]. As suggested by comparative studies, the numerous ERV lineages found in modern mammal genomes arose from multiple independent events of genome invasion, also affecting the host germline, followed by the vertical inheritance of ERVs as host alleles. As a relevant number of such events occurred after the divergence of mammalian orders, each mammalian order tends to have its own distinct ERV content, composition and history, with some ERVs being unique even to individual genera or species, and the same diversification trend also applies to vertebrates as a whole [51,52]. In the case of primates, for example, lineage-specific ERV insertions have been observed in the genomes of African great apes that are absent from human and Asian ape genomes [53]. Even though the retrotransposition activity of human ERVs (HERVs) is presently very limited or absent [54], there is growing evidence that HERVs are widely expressed in human tissues, even in the absence of protein production, which has led to an intense study of their possible roles in human pathologies, including cancer, autoimmune disorders and infectious diseases [55,56,57].

ERVs are usually divided into three classes based on their affinity to exogenous animal viruses: class I (gammaretrovirus- and epsilonretrovirus-like), class II (betaretrovirus-like) and class III (spumaretrovirus-like). Concerning individual ERV group classification, it is still incomplete—also due to the relatively recent availability of assembled genome sequences for many vertebrates—and sometimes controversial, given that ERVs are not always named based on phylogenetic and taxonomical criteria. A recent work performed on the human genome with the software RetroTector employed a multi-step classification approach, identifying ~3300 reasonably intact HERV loci that were divided in 31 taxonomical groups, plus 39 “non-canonical” clades showing high degrees of mosaicism and recombination events [58]. Such a comprehensive genomic analysis, complemented by the available detailed characterizations of individual HERV groups (see, for instance, [59,60,61,62]), represent an ideal background to evaluate HERV expression in human tissues and its variation in diseased contexts [63].

The high proportion of retrotransposons in mammalian genomes, exceeding 90% of all TEs in humans and 95% in mice and rats, together with the presence of at least one family of currently accumulating retrotransposons in most mammals [38], has attracted the greatest attention onto this TE class. An impressive body of studies in the last two decades have addressed the role that retrotransposons played in mammalian, and particularly in human, evolution by facilitating the appearance of genomic novelties. As new evidence accumulated, authoritative reviews were published at various times, covering in great detail the genomic impact and the different emerging aspects of retrotransposons [6,8,13,37,38,52,54,64,65,66,67,68,69,70,71,72,73,74,75,76,77,78]. In a nutshell, it is thought that retrotransposons contributed to the generation of genomic novelties in two main ways: (i) indirectly, through the promotion of genomic rearrangements; and (ii) directly, through exaptation of retrotransposon-derived sequences.

### 2.2. Retrotransposons as Drivers of Genomic Rearrangements

Their indirect contributions to genomic novelties derive from the fact that retrotransposons, due to their high copy number and high sequence homology within families, are a relatively frequent substrate of unequal recombination events producing gene and/or exon duplication, shuffling or deletion. As an additional mechanism, retrotransposons sometimes carry flanking genomic sequences with them (a process referred to as 5’ or 3’ transduction) thus potentially introducing new copies of genes/exons into new locations [54]. The generation of retrogenes is a further indirect consequence of the presence of autonomous retrotransposons, the machinery of which may be exploited by mRNAs or other RNAs to generate new copies of their coding sequences [7]. Independently from the mechanisms of their generation, new gene copies have great potential for neofunctionalization favoring phenotypic evolution [79], a property that, contrary to what has been prevailingly thought, is likely to also apply to pseudogenes and retropseudogenes [80].

### 2.3. Retrotransposon Exaptation as a Source of Genomic Novelties

The exaptation of retrotransposed sequences, consisting in their cooption for a current function out of a hitherto neutral evolution mode, is a well-documented phenomenon [2,74]. In general, large-scale DNA editing of retrotransposons, by simultaneously generating large numbers of mutations, may have accelerated their exaptation during mammalian evolution [81]. In a similar vein, inverted SINE repeats being part of longer RNAs may have promoted RNA editing by adenosine to inosine deamination, thus generating potential novelties in both coding and regulatory sequences [82].

For simplicity, two major exaptation modes can be distinguished. According to the first mode, retrotransposon-derived sequences become physical and functional parts of transcription products, even being eventually translated into protein sequences. The second mode consists in the co-optation of retrotransposon-derived sequences as transcription regulatory elements or 3D genome boundary elements. This exaptation mode, allowing retrotransposon sequences to exert their influence without becoming incorporated into gene products, might have had an even wider influence on genome evolution [83,84].

#### 2.3.1. Retrotransposon-Derived Sequences within Gene Products

As to the first mode of action, there is solid evidence that SINE (in particular, primate-specific Alu) exonization contributes to both untranslated and protein-coding regions of mRNAs [85], as well as portions of long noncoding RNAs [86], to which the embedded Alu can confer new regulatory functions [87]. Alu-derived exons are often the site of alternative splicing, due to the presence in the Alu body of multiple cryptic splice sites [88]. SVA-mediated transduction events, involving alternative mRNA splicing at cryptic splice sites, have been found to promote exon shuffling and thus genomic novelty [89]. At the same time, cells have evolved precise mechanisms to control Alu and the incorporation of other retroelements within mRNA sequences via their cryptic splice sites, as their incorrect presence might induce devastating physiological responses [90]. Moreover, exonic SINE sequences embedded into the 3’ UTR of mRNAs participate in different layers of post-transcriptional gene regulation, which may also involve intermolecular base-pairing with SINE sequences embedded in lncRNAs [43,88,91,92]. Alu SINEs embedded into precursor transcripts were also found to promote the formation of circRNAs [93], a complex family of eukaryotic regulatory transcripts under intense study [94,95]. There is also abundant evidence for TE-derived microRNAs, some of which are potentially involved in human evolution and disease [96,97,98,99,100].

In the case of autonomous retrotransposons, which contain protein-coding sequences in their body, several striking cases of exaptation of retrotransposon-encoded proteins as new host proteins have been documented. A remarkable example is represented by syncytins, an ensemble of Env proteins coded by different ERVs in the genome of various vertebrates, that through a process of convergent evolution led to the development of the placenta in eutherian mammals [55,101]. In fact, the union between maternal and fetal cells to constitute the placental syncytiotrophoblast—the main site of trophic exchanges during pregnancy—is mediated by the fusogenic activity of syncytins, which changed from being mechanisms of viral entry to exerting physiological activity domesticated to serving the host biology [55]. Some syncytins are indeed thought to have a role in other placenta-associated functions, such as the establishment of maternal immune-tolerance against the fetal allograft through their natural immune-suppressive properties, which in ancestral infections likely guaranteed their immune escape [102,103].

#### 2.3.2. Retrotransposons as a Source of *cis*-Regulatory Sequences

**Figure 2:**
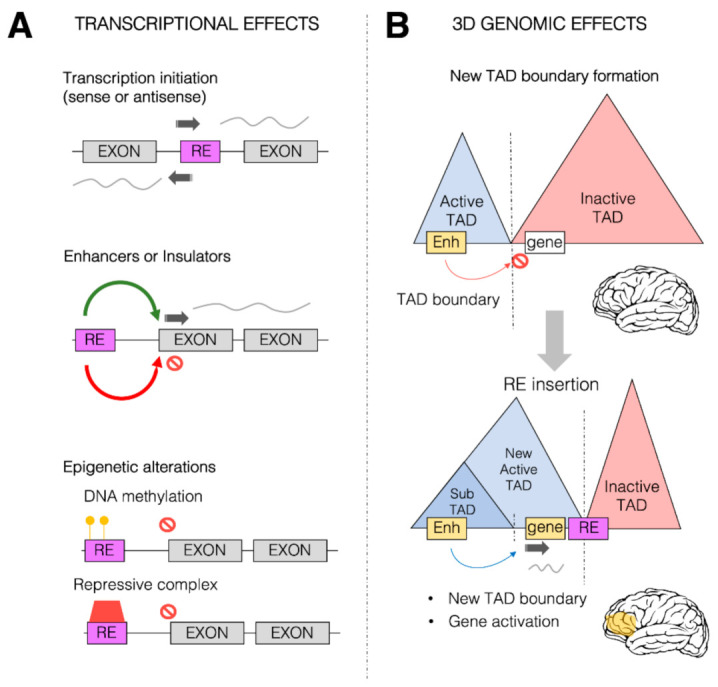
Transcriptional and 3D genome effects of retrotransposons. Retrotransposons (or retroelements, RE) are responsible for a wide range of possible effects on both transcription control and 3D genome organization. (**A**) Schematic representation of some of the predominant effects of REs on transcription. REs (purple box) can be inserted within the coding region between two exons (gray boxes), providing new transcription start sites (TSS, dark gray arrow) for both sense and antisense transcription. REs can also provide new *cis*-regulatory sequences (such as enhancers or insulators) which can in turn activate (green arrow) and repress (red arrow) transcription of the associated gene. REs could also alter the epigenetic state of a given gene, leading to its transcriptional repression, by increasing the DNA methylation (yellow circles) within the promoter region of the transcription unit and directly or indirectly recruiting repressive complexes (red box). (**B**) REs can impact the 3D genome organization of the chromatin within the nuclei. REs (especially Alu elements) are found to be enriched at topologically associating domain (TAD) boundaries. Represented in the Figure is a putative case in which two TADs, one active (blue) and one inactive (red), are separated by a TAD boundary. This boundary limits the action of a brain enhancer region (yellow box) within the active TAD towards a gene (white box) within the inactive TAD, thereby impeding the ectopic brain expression of the gene. As a result of an RE insertion event within the inactive TAD, the 3D genome organization is altered, and a new active TAD is formed due to the boundary effect of the RE. This leads to the spreading of the active TAD over the gene, which allows the brain enhancer region (yellow) to now induce gene activation and therefore its ectopic expression within the brain (yellow area).

Given the centrality of *cis*-regulatory elements, and particularly of enhancers, in orchestrating organ-, tissue- and cell type-specific gene expression both during development and in adult organisms [104], it has been argued that the “vast majority of the genetic changes responsible for the evolution of morphology occur at pre-existing *cis*-regulatory elements” [105], and that TE-mediated *cis*-regulatory network rewiring has been one of the key mechanisms for the appearance of such changes [6]. In the last 10–15 years, the exaptation of TE-derived sequences (especially retrotransposon-derived) as *cis*-regulatory elements has been well documented by a rapidly growing body of studies, the majority of which have focused on mammalian genomes, characterized by the overwhelming prevalence, in terms of both amount and activity, of retrotransposons over DNA transposons. Retrotransposon-derived *cis*-regulatory sequences have been reported to play several roles in gene regulation as promoters, enhancers, silencers and boundary elements [2,83]. In general, due to their own replicative needs, retrotransposons have evolved *cis*-acting sequences mimicking those of the host, a fact that predisposes them to *cis*-regulatory activity [76]. Although we are still far from a comprehensive picture of the multiple layers of TE-derived regulatory novelties and their integration with the whole genomic background of mammalian evolution, various *cis*-regulatory modes of TE exaptation have begun to be clearly portrayed (Figure 2).

First of all, many binding sites for diverse TFs are contributed by retrotransposons, as mainly revealed by genome-wide TF occupancy mapping by chromatin immunoprecipitation coupled with high throughput sequencing (ChIP-seq) [106]. Although some of the TF binding sites carried by TEs are justified by their need to employ host TFs for their own life cycle, others may have been acquired independently through TE propagation mechanisms [34]. Molecular evolution studies have revealed waves of expansion of the TF target repertoire over the course of vertebrate evolution, with TEs majorly contributing to such expansions [107]. TFs tend to bind to TE-provided cognate sites in a species-specific manner, in line with the expansion of different TE subfamilies at different evolutionary timepoints [83]. A striking example of how the evolutionary recruitment of TE-derived TF binding contributed to mammalian evolution is provided by the TE-dependent transformation of the uterine regulatory landscape in the evolution of mammalian pregnancy [108]. An emerging topic that is potentially highly relevant to the exaptation of TE-binding TFs, is that of Krüppel-associated box domain zinc finger proteins (KRAB-ZFPs). The great expansion and diversification in mammals of these TFs has been correlated with the invasion of new endogenous retroelements, which require specialized mechanisms of repression via the binding of specific KRAB-ZPs and subsequent recruitment of the KAP1 corepressor [28]. It is thought that the arms race between KRAB-ZFPs and their target retroelements, facilitated by the evolutionary plasticity conferred on both contenders by the repetitive organization of their genes, favored retroelement domestication, allowing them to develop *cis*-regulatory functions, to which KRAB-ZFPs have the potential to directly contribute as enhancers or promoter-binding TFs [28,71,109,110].

A second, more complex mode of TE exaptation for *cis*-regulatory purposes is represented by TE-derived clusters of TF binding sites, exemplified by the contribution of species-specific, composite enhancers to mouse placental development by rodent endogenous retroviruses [111]. In addition, mouse-specific LTRs have been found to carry multiple pluripotency TF-binding sites (specifically, ESRRB-, KLF4- and SOX2-binding motifs) regulating gene expression in a mouse embryonic stem cell (ESC)-specific manner, thereby distinguishing ESCs in mice from ESCs in other species [112]. In a similar vein, recent hominoid-specific LTR and SVA retrotransposons were shown to host enhancers that were active in human naive ESCs and embryonic genome activation [110]. Systematic studies of TEs’ contribution to enhancer function have benefited greatly from high-resolution profiling of the regulatory epigenome, such as the profiling of DNase hypersensitivity, histone H3-lysine 4 mono-methylation (H3K4me1) and histone H3-lysine 27 acetylation (H3K27ac) as typical enhancer chromatin signatures [113] and by the use of a chromatin characterization software such as ChromHMM [114]. A recent comprehensive quantification of the epigenomic status of TEs across many human tissues and cell types revealed that approximately one quarter of the human regulatory epigenome is composed of retrotransposed sequences, with motif-enriched LTRs being particularly favorable substrates for the evolution of new host regulatory elements [115]. In other studies, based on epigenomic profiling, evolutionary novelties in primate gene regulation were similarly found to have TEs as the primary source, with a major contribution from ERV-derived sequences [116,117]. Accordingly, a subset of ERV sequences were found to be significantly enriched in *cis*-regulatory elements, having a critical role in primate liver gene regulation [117]. A fascinating example of ERV contribution in the shaping of entire regulatory pathways is represented by the interferon (IFN) transcriptional network, a crucial innate antiviral system which also serves as a fundamental effector to initiate and maintain adaptive immunity. Chuong and coauthors showed that ERV insertions had a central role in its evolution and amplification, accounting for the independent dissemination of a wide number of IFN-inducible enhancers in many mammalian genomes, which are required for the correct functioning of different immune responses [118]. A similar scenario is found for p53 tumor suppressor factor, of which the genomic binding sites in humans overlap in more than one-third of cases with ERV elements [119]. Of note, these binding sites are primate-specific and not present in other mammals, further demonstrating that TEs are able to shape important regulatory networks in a species-specific manner. An intriguing observation, consistent with the previous ones, is that of the pervasive function of an ape-specific class of ERV-derived LTRs, LTR5HS, as early embryonic enhancers, regulating hundreds of human genes [120], and the strong contribution of ERV and L1 retrotransposon families to species-specific differences in enhancer activity between chimpanzee and human cranial neural crest cells [83,121]. Epigenome profiling also allowed researchers to distinguish between older retrotransposon copies displaying most of the features of de facto enhancers and younger copies that seem instead to be configured as proto-enhancers, serving as a repertoire for the de novo evolutionary birth of enhancers [122]. Despite the scarcity of studies, an intriguing retrotransposon feature favoring their exaptation as enhancers is their intrinsic capability of generating functional non-protein-coding RNAs (ncRNAs) that could overlap with the so-called enhancer RNAs (eRNAs) [123], thereby raising the possibility that many eRNAs could be generated through TE-derived ncRNAs.

#### 2.3.3. Involvement of Retrotransposons in Three-Dimensional Genome Architecture

Chromosome contacts within the nuclear space, recently revealed at unprecedented resolution by HiC and complementary approaches [124], exert a wide and still largely unexplored influence on gene regulation by demarcating regulatory districts in a highly dynamic way. At a large scale within nuclei, chromosomes segregate into regions of preferential long-range interactions that form two mutually excluded types of chromatin, referred to as “A” and “B” compartments [125], the formation of which has been recently linked to homotypic clustering of L1 and B1/Alu, respectively [126]. At a scale of tens to hundreds of kilobases, chromosomes fold into domains with preferential intradomain interactions known as topologically associating domains (TADs), which harbor the potential to influence enhancer function and thus gene regulatory networks [127,128,129,130,131,132]. TAD demarcation is achieved by specific regions called TAD boundaries, which are enriched for the occupancy of CCCTC-binding factor (CTCF), a zinc finger DNA binding protein also known to mediate the formation of chromatin loops [133]. SINE retrotransposons have also been found to be enriched at TAD boundaries [134,135]. Curiously, in rodents (but not in humans) B2 SINE retrotransposons have been shown to carry CTCF binding motifs, and therefore rodent B2 SINEs can contribute to clustered CTCF sites at TAD boundaries, thus helping in the maintenance of genome organization [136]. However, the rapid expansion of rodent SINEs might provide excessive CTCF sites throughout the genome, therefore critically increasing the possibility of genome mis-folding due to the creation of aberrant CTCF sites. In this context, a complex formed by CHD4, ADNP and HP1 chromatin proteins (ChAHP complex) has been shown to play a role in the maintenance of evolutionarily conserved spatial chromatin organization via the buffering of novel CTCF binding sites that emerge through SINE expansion [137]. Moreover, SINE and other retrotransposons have been proposed to participate in the establishment of species-specific chromatin loops by introducing novel binding sites for architectural proteins, including CTCF [138]. CTCF might also participate, together with other proteins, in the DNA methylation and histone modification boundary activity recently attributed to currently active copies of mouse B2 SINEs, which might be involved in the epigenomic and phenotypic diversification of mouse species [139].

The contribution of retrotransposons to chromatin regulatory domains is not limited to providing CTCF binding clusters. MIR retrotransposons, for example, have been shown to provide regulatory sequences, functioning as insulators in the human genome independently from CTCF [140]. The presence of binding sites for the multi-subunit DNA binding protein TFIIIC is a distinguishing feature of SINEs, and TFIIIC bound to Alu elements has been shown to influence gene regulation through its chromatin looping and histone acetylation capacities [141,142]. In the case of SINEs exapted as enhancers or TAD boundaries, their regulatory function might even take advantage of their Pol III-dependent transcription, which was recently demonstrated to occur with a marked cell-type specificity [123,143,144]. Retrotransposon transcription has also been shown to be required for the cell type- and species-specific chromatin architecture remodeling properties recently attributed to the primate-specific HERV-H TE family of LTR retrotransposons [145].

## 3. Genomic Sources of Evolutionary Novelties in the Mammalian Brain

A unique feature of the mammalian brain, distinguishing mammals from all other vertebrates, is the presence of a six-layered cerebral cortex (neocortex) representing an arrangement of telencephalic neurons that is absent from even the closest vertebrates. The issue of how such a novelty originated, as well as of how exclusively it is responsible for the functional peculiarities of the mammalian telencephalon, are still largely undecided [146]. On the one hand, the appearance of a telencephalic neuroanatomical structure without any homologous structure in non-mammalian vertebrates deserves the utmost consideration. On the other hand, functionally relevant homologies between vertebrate telencephala may occur beyond the neuroanatomical level. In particular, classical and recent evidence suggests that the core neuronal cell types participating in neocortical circuits are shared across birds, reptiles and mammals [147].

A striking feature distinguishing eutherian (e.g., mice and humans) from non-eutherian (e.g., marsupials and monotremes) mammals is the presence in the former of the corpus callosum as a way to connect the neocortical hemispheres. During mouse and human cortical development, the transcription factor SATB homeobox 2 (SATB2) specifies neurons projecting via the corpus callosum, whereas another transcription factor, BCL11B/CTIP2, appears to specify neurons that project subcerebrally. In a recent comparative study, it has been shown that differential timing in the expression of SATB2 is critical for different neuronal projection fate in eutherian (mouse) and non-eutherian (dunnart) mammals [148].

A feature whose variation across mammals has received particular attention is brain size. Among vertebrates, both birds and mammals generally evolved larger brains relative to body size [146]. Such an evolutionary increase in relative brain size tends to be associated with increased numbers of neurons in the telencephalon [149], which is dominated by the neocortex in mammals. A large neocortex is not an invariant feature of all mammals, however. According to comparative analyses, expansion and contraction of the neocortical surface area occurred independently numerous times across mammalian phylogeny. In particular, primates are characterized by an increase in the neocortex with the maintenance of high neuron packing density, and the human neocortex is more enlarged and elaborated than any other primate’s brain structure [150,151]. The expanded human cerebral cortex is also intricately folded, even though gyrencephaly is likely to be an evolutionarily ancient trait present in the common mammal progenitor [152]. The increase in size of the human neocortex also entailed an increase in the number of neocortical areas with respect to the 15–25 neocortical areas thought to be shared across mammals [150,153].

More than the absolute or relative size or the number of neurons or glial cells, the key to the human brain’s unique capacities is likely to be represented by an enrichment of the repertoire of neural cell types and by wider and more elaborate patterns of neuronal connectivity. Such features are made possible by some developmental peculiarities of the human brain. One of them is its prolonged developmental course, during which expanded proliferative zones with neural stem and progenitor cells with enhanced proliferative capacities facilitate neocortex expansion [154,155].

Understandably, a vast array of studies has been devoted to linking the development of human brain evolutionary specialization to specific genetic changes and related molecular/cellular mechanisms [155,156]. Genomic innovations thought to have contributed to human neocortex structural and functional novelties, in particular through the enhancement of neurogenesis and/or synaptogenesis, are mainly represented by human-specific gene duplications (HSGDs) and mutations to non-protein coding regulatory regions.

As to HSGDs, a recently discovered example is represented by Notch homolog 2 *N*-terminal-like (*NOTCH2NL*) gene duplication, producing human-specific NOTCH2 paralog proteins that enhance neural progenitor proliferation [157,158,159]. In other studies, SLIT-ROBO Rho GTPase activating protein 2 (*SRGAP2*) duplications were found to favor human-specific traits of synaptic development, such as protracted synaptic maturation [160,161,162]. Another HSGD that is likely involved in neurodevelopment is Rho GTPase activating protein 11B (*ARHGAP11B*), the product of which promotes basal progenitor amplification and neocortex expansion and folding [163,164,165].

As to noncoding regions with regulatory roles, much effort has been devoted to the identification of human-specific changes in *cis*-regulatory regions that are likely to cause human-specific patterns of gene expression involved in brain development and function [154]. Support for the evolutionary importance of such changes has come from studies showing that the regulatory regions of neurodevelopmental genes were particularly prone to positive selection [166,167]. Many of the regulatory regions whose evolution was found to be accelerated in humans display typical features of enhancers, a large proportion of which are active in the brain [168,169,170]. A few described cases of human-specific changes in gene expression patterns that are important for brain development include those affecting the enhancers of neuronal PAS domain protein 3 (*NPAS3*), encoding a TF involved in neurogenesis [171], frizzled class receptor 8 (*FZD8*), coding for a Wnt protein receptor involved in neocortex development [172], osteocrin (*OSTN*), encoding an activity-dependent secreted factor [173], cut-like homeobox 1 (*CUX1*), encoding a TF involved in dendritic development and implicated in autism spectrum disorder [170], and fibroblast growth factor receptor 2 (*FGFR2*) [174]. Enhancer–promoter interactions, that are key to the implementation of gene-regulatory programs, take place in the context of a complex and dynamic 3D chromatin architecture, of which the involvement in brain development, neuronal activity and complex brain disorders is only starting to be appreciated [175,176]. Based on these premises, it is not unexpected that brain evolutionary innovations have occurred through the 3D rewiring of the enhancer–promoter interactome, as very recently revealed for primate corticogenesis [14].

Finally, it should be noted that genomic novelties affecting brain development/function might do so by generating novel ncRNAs. This appears to be the case for the product of the highly accelerated region 1A (*HAR1A*) gene, originally identified as one of the most rapidly evolved non-protein coding regions in humans [166,177]. More generally, it has been suggested that the expansion of ncRNA inventories, in particular those of miRNAs, played a role in the emergence of vertebrates’ morphological complexity [178]. The developmental profiles of miRNAs were found to display a fast rate of human-specific evolutionary change, and to drive gene expression changes in the human prefrontal cortex [179]. In the last decade, further evidence has been accumulating in support of the notion that miRNAs can accelerate the evolution of the human brain by introducing subtle alterations in gene expression patterns [180]. Other attractive candidates for such a role are long ncRNAs (lncRNAs), characterized by a remarkable diversity of gene regulatory activities. Although mechanistic studies on lncRNAs’ evolutionary impact are still in their infancy, it is very telling that of the tens of thousands of lncRNAs encoded by mammalian genomes, as many as 40% are expressed specifically in the brain, and thousands of new lncRNAs have appeared during primate nervous system evolution [181,182].

Given the fundamental role played by retrotransposons in phenotype-impacting genomic innovation in mammals and other vertebrates, the growing evidence of retrotransposon involvement in mammalian brain evolution is not surprising. What makes this evolutionary mechanism particularly noteworthy for the brain is that it is one of the human tissues in which somatic retrotransposition has been found to occur considerably at some stage in development, leading to the suggestion of a key role of the mobilome in the expansion of higher brain functions in modern humans and, as a downside, in their proneness to age-related neurodegeneration [16,71,183]. The growing body of evidence in favor of retrotransposons as drivers of brain evolution in mammals deserves a specific and detailed discussion, which will be presented in the following sections.

## 4. Contribution of Non-LTR Retrotransposons to Mammalian Brain Evolution

### 4.1. Contribution through SINE Exaptation

In most mammals, SINEs account for >10% of the genome, and their lineage-specific diversification significantly contributes to the distinctive genome composition and arrangement of the different mammalian lineages [13]. As discussed above (Section 2), SINE exaptation has greatly contributed to genomic innovations in mammals, with important repercussions on brain evolution. From a historical perspective, the first discovered case of SINE exaptation affecting mammalian brain function is represented by the primate-specific BC200 RNA [184], a brain-specific ~200 nt-long ncRNA originated from an Alu monomer sequence and playing a regulatory role in dendritic translation [185]. BC200 RNA dysregulation has been associated with neurodegeneration, but also with neoplastic changes in various tissues [186]. Curiously, the mouse gene *Bc1*, coding for brain-specific BC1 RNA, the rodent functional counterpart of BC200 RNA, is not itself a SINE, but it has been shown to be the master gene from which the murine ID SINE subfamily originated [184]. Although there are no other such well-characterized cases of SINE-derived RNAs involved in brain function, other Alu-related transcripts represent interesting candidates [187]. In particular, the Alu-derived human NDM29 transcript induces a neuron-like phenotype when transfected into undifferentiated neuroblastoma cells [188], and members of the snaR family of ncRNAs, with a possible role in translation, were found to be differentially expressed in different brain regions [189]. Given the recent improvement of methods of detecting and quantifying the expression of individual SINE loci in cells and tissues [144,190,191,192], including single neurons [193], it is likely that more cases will be revealed of exapted SINEs producing ncRNAs involved in brain function.

Evolutionary novelties might also have occurred due to the regulatory effect that SINE sequences can exert on longer RNAs (either mRNAs or lncRNAs) in which they are embedded. In particular, the aforementioned ADAR-dependent A-to-I RNA editing (see Section 2.3), which has been shown to be widely promoted by Alu inverted repeats in primate transcripts [82], is thought to be critical for brain development and functions, including their alterations in neurological and neurodegenerative disorders [18,194,195]. Exonized Alus within the 3’ UTR have regulatory potential if targeted by miRNAs, as recently shown for a primate-specific isoform of the cytochrome P450 family 20 subfamily A member 1 (*CYP20A1*) mRNA, whose 3’UTR includes numerous miRNA-targeted Alu sequences acting as miRNA sponges with neuron-specific effects [196]. Embedded SINEs have also been shown to confer translation regulatory potential to a class of antisense lncRNAs, called SINEUPs, described both in mice and in humans. In SINEUP lncRNAs, a 5’ sequence specifically targets an mRNA, whereas an inverted embedded SINE sequence, bound by the RNA binding protein ILF3, confers translation-enhancing activity [197]. Remarkably, the first described SINEUP lncRNA is antisense to (and upregulates the translation of) the mouse ubiquitin carboxy-terminal hydrolase L1 (*uchl1*) mRNA, whose product is essential for neuron maintenance and brain function [198]. Many more natural antisense transcripts with potential SINEUP functions have been identified in the human brain transcriptome [199], again suggesting that retrotransposons’ impact on brain evolution and function is played out on multiple, largely unexplored layers.

Perhaps the most substantial set of evidence supporting a role of SINE exaptation in brain evolution points to SINEs as facilitators of *cis*-regulatory evolution [13]. Early studies showed that Pax6, a transcription factor with a key role in central nervous system development, has binding sites in specific Alu elements in humans [200] and in a subset of B1 SINEs in mice [201]. These SINE-derived binding sites are not evolutionarily related in the two species, thus suggesting that SINE-dependent diversification of gene regulatory networks is involved in neurodevelopment. The idea that retrotransposon exaptation as enhancers contributed to mammalian brain novelties received strong support from a series of studies published in the second half of the 2000s, inspired in part by the observation that a significant fraction of evolutionarily conserved non-protein-coding sequences in mammals, probably involved in mammal ontogeny as cis-regulatory elements, overlaps with retrotransposons [13]. In particular, through independent studies combining computational homology searches and assays of enhancer function, it was shown that members of two newly identified SINE families, named AmnSINE1 and LF-SINE, and a member of the previously described MIR (or CORE-SINE) superfamily underwent exaptation as distal *cis*-regulatory elements of genes involved in nervous system development and function in mammals [202,203,204]. Specifically, an enhancer of the ISL1 gene, encoding a TF involved in motor neuron differentiation, was found to be constituted by an LF-SINE (classifiable as SINE2/tRNA according to Repbase [29]) which might have been exapted before tetrapod divergence [202]. Even more strictly related to mammalian brain evolution, the neuronal enhancer nPE2 of the proopiomelanocortin (*POMC*) gene was found to originate from the exaptation of a MIR (SINE2/tRNA) retrotransposon in the lineage leading to mammals [203], and two AmnSINE1 elements (classifiable as SINE3/5S [29]) were shown to constitute enhancers for *FGF8* and *SATB2* genes, of which the products control different aspects of forebrain development in a mammalian-specific manner [204]. In particular, the AmnSINE1-derived enhancer referred to as the AS071 locus controls FGF8 expression in the diencephalon and the hypothalamus, thus allowing for FGF8-dependent, mammalian-specific patterning of the forebrain. AmnSINE1 at the AS021 locus, highly conserved across mammalian species, likewise functions as an enhancer, whose activity recapitulates the expression pattern of Satb2, a sequence-specific DNA binding protein involved in transcription regulation and chromatin remodeling and required for mammalian neocortex development. Remarkably, the AS021 SINE enhancer was later shown in mice to drive the expression of SATB2 in a subpopulation of callosal neurons, connecting the two hemispheres of the cerebral cortex via the corpus callosum, a eutherian-specific brain structure [205]. A further in-depth study of the organization of the mouse AS071 enhancer revealed a modular structure with functionally distinct sub-elements cooperatively participating in enhancer activity in three distinct diencephalic domains, with the AmnSINE1 sub-element specifying the enhancer activity to the ventral line of the hypothalamus [206]. Overall, AmnSINE1 retrotransposons are thought to have played a relevant role in the evolutionary emergence of mammals [69], an idea further corroborated by the discovery that AmnSINE1 constitutes, together with other TE-derived sequences, an enhancer module involved in morphogenesis of the mammalian secondary palate through the control of wnt5a expression [207].

The integrated contribution of SINEs and other retrotransposons to *cis*-regulatory novelties by convergent evolution finds a striking example in the aforementioned enhancers of *POMC*, a gene expressed in mammalian neurons of the hypothalamus arcuate nucleus. An in-depth genome sequence comparison of different vertebrates and mammals, together with enhancer assays in transgenic mice, revealed that in addition to the MIR-derived nPE2 enhancer, neuron-specific *POMC* expression also involves another enhancer element, nPE1, originating from the exaptation of an LTR retrotransposon before the placental mammal radiation [208]. An extreme example of TEs’ contribution to *cis*-regulatory elements is represented by the gene *NPAS3* (neuronal PAS domain-containing protein 3), coding for a transcription factor involved in both mouse and human brain development, as well as in psychiatric illness [209]. *NPAS3* is the human gene containing the largest number of genomic regions showing accelerated evolution in the human lineage, also referred to as human-accelerated non-protein-coding elements (HAEs) [171]. One of these elements, referred to as 2xHAR142 and located in the fifth intron of *NPAS3*, was shown to behave as a transcriptional enhancer that may have contributed to a uniquely human *NPAS3* expression pattern. Intriguingly, 2xHAR142 contains sequences derived from an MIR retrotransposon, and other SINEs and LINEs may have contributed to other HAEs associated with NPAS3 [210].

A largely unexplored layer of the potential involvement of SINEs (and other retrotransposons) in brain evolution, which has only recently begun to be glimpsed, is related to their involvement in three-dimensional genome architecture (see Section 2.3.3). Specifically, high-resolution mapping of the genome architecture of the developing macaque brain, together with cross-species 3D genome analyses, recently revealed human-gained TAD boundaries enriched in evolutionarily young TEs, including Alu, LINE-1, ERV1 and ERVK retrotransposons [14]. Such boundaries tend to be more enriched in brain-development-related genes, with implications for the appearance of human-specific brain properties. For example, the human-gained TAD boundary around contactin 5 (*CNTN5*), a gene involved in neuron circuit formation and autism spectrum disorders, contains Alu Y elements and is correlated with increased *CNTN5* expression in humans compared to macaques [14].

### 4.2. Contribution through SINE-Dependent Genomic Rearrangements

Apart from having evolutionary roles through exaptation, SINEs most likely also contributed to nervous system evolution by favoring gene duplication events. Particularly relevant to this issue is the enrichment of segmental duplications in primate genomes compared with other mammals. Segmental duplications are thought to have created novel primate gene families, thus potentially driving primate-specific evolutionary changes and contributing to human genic and phenotypic variation [211]. Remarkably, among the mechanisms of segmental duplication, an important role was likely played by nonallelic homologous recombination among Alu repeats, of which a burst of activity during a narrow window of primate evolution provided a myriad of nearly identical sites favoring this kind of recombination event [212]. An example of how this phenomenon may have contributed to brain evolution is provided by the evolution of human-specific *SRGAP2* genes through incomplete segmental duplication, an event most likely favored by Alu elements mapping precisely at duplicated segment boundaries [160].

Recombination between Alu elements can also result in genomic deletions, with the potential both to contribute to human genetic disorders and to introduce genomic novelties of potential evolutionary relevance [213]. An example of Alu recombination-mediated deletion potentially affecting the evolution of typically human nervous system features is represented by the loss, in humans compared to chimpanzees, of the fourth exon of the cholinergic receptor nicotinic alpha 9 subunit (*CHRNA9*) gene, contributing to distinctive olfactory and auditory traits between these two primates [213]. Several genes associated with neurological and neurodegenerative disorders have been reported to be susceptible to deleterious Alu-mediated rearrangements (reviewed in [18]).

### 4.3. Contribution of LINEs

It was widely believed that LINE-1 retrotransposition could occur only in germ cells due to their potential to contribute to the expansion of these TEs in subsequent generations. However, this hypothesis was drastically changed by the finding that differentiation of adult rat hippocampal neural stem cells into neuronal precursor cells (NPCs) and neurons is accompanied by an increase in LINE-1 transcript abundance, and the finding that engineered LINE-1 could retrotranspose in cultured NPCs and in the brain of transgenic mice [214,215]. Subsequent studies also revealed that engineered LINE-1 could retrotranspose in both fetal and human embryonic stem cell (hESC)-derived NPCs [216] and that engineered human LINE-1 showed enhanced somatic retrotransposition in neurons of mouse models lacking the methyl-CpG-binding protein 2 (MeCP2) [217] and in human neural stem cells lacking the ataxia telangiectasia mutated (ATM) kinase protein [218]. Further insights into LINE-1 retrotransposition in the brain came from the development of specific techniques for retrotransposon-capture and sequencing (RC-seq), which unveiled how endogenous LINE-1 retrotransposition could be accountable for somatic mosaicism in the human brain [219]. More sophisticated single cell-based genomic approaches have provided key insights regarding the frequency of neuronal retrotransposition, with the frequency estimated to be <0.6 insertions per cell [220]. Very recently, upregulation of evolutionarily young LINE-1 elements (but not of other retrotransposons) was found to occur genome-wide in *DNMT1* KO-derived NPCs, and to affect the expression of L1-controlled genes involved in neurodevelopment [221]. More studies will be needed to determine if the LINE-1 retrotransposition rate could vary depending on different brain regions and to explain why NPCs tend to be permissive to LINE-1 retrotransposition.

LINE-1 can therefore retrotranspose in the human brain, and the outcomes and the consequences of this remain largely elusive and open to further investigation. The more of the functional importance of neuron somatic retrotransposition is revealed, the more relevant it will be to clarify its evolutionary origins. At the same time, new scenarios of LINE *cis*-regulatory exaptation are just starting to appear, revealing its effect on the generation of morphological novelties in mammals, including 3D genome innovations during primate corticogenesis [14,222] and the very recently reported contribution of L1 to the tissue-specific (including brain-specific) *cis*-regulatory landscape across mammalian lineages, spanning more than 150 million years of mammalian evolution [223].

## 5. Contribution of LTR Retrotransposons to Mammalian Brain Evolution

ERV expression has been investigated in the brains of mammals. Concerning the human brain, this field of study is also highly relevant due to the possible role of HERV products in different neuroinflammatory, neurodegenerative and neuropsychiatric disorders, such as multiple sclerosis, amyotrophic lateral sclerosis and schizophrenia, as reviewed elsewhere [224,225,226].

### 5.1. ERV Contribution to Mouse Brain Development and Physiology

Currently, most of the direct information regarding the contribution of ERV to mammalian brain development is derived from studies in mouse models focused on the expression of individual ERVs, as well as their shaping of entire transcriptional patterns. At the protein level, the mouse genome presents a MuLV-ERV locus with full coding potential on chromosome 8 that has shown brain expression limited to the cerebellum, in which its low methylation status was unique as compared to the other brain regions. Apart from being cerebellum-specific, MuLV-ERV_mch8_ expression was also age-dependent, with almost no expression at 2 weeks and a plateau at 6 weeks [227]. This, together with the fact that the MuLV-ERV locus is integrated into a region surrounded by genes linked to neuronal development and/or inflammation, might indicate the involvement of MuLV-ERV_mch8_ in cerebellar biology. However, to date, the actual role of this ERV remains to be fully elucidated.

Moving to ERV transcriptional regulation, it is known that in most organs ERVs are transcriptionally silenced during early embryogenesis by histone and DNA methylation, showing a striking shift in their transcriptional activity after the first few days and according to cell differentiation [228]. In line with this, the tripartite motif-containing protein 28 (TRIM28, also known as KAP1)—essential for early development in mice—forms a complex on ERV LTRs and mediates their silencing in the first few days of embryogenesis through histone 3 lysine 9 trimethylation (H3K9me3) [229]. Then, in mouse embryonic stem cells and early embryos, this TRIM28-mediated silencing mechanism is replaced by DNA hypermethylation of the LTRs, leaving the transcription of ERV sequences unaltered even when TRIM28 is experimentally deleted [228]. This is not true in the brain, however, where TRIM28-mediated control is used to dynamically regulate the transcription and silencing of ERVs. In fact, the deletion of TRIM28 from NPCs is followed by a marked increase in ERV transcription, sustained especially by selected members of two ERV groups, MMERVK10C and IAP, which are not subjected to DNA methylation [230]. Accordingly, the proportion of unmethylated DNA in NPCs is higher than in somatic cells. Of further note, unmethylated ERVs in NPCs are often integrated near to coding regions, and the lack of silencing at their LTRs makes them transcriptional start sites for these neighboring genes—but also for lncRNAs when found in gene-free regions—suggesting a central role in the control of gene networks in the mouse brain [229]. Such a role might be played also in human NPCs, given that disruption of TRIM28 in the mouse brain leads to behavioral alterations that are comparable to the ones observed in certain psychiatric disorders [228]. Considering the emotional spectrum, ERV expression is known to be influenced by stressful conditions as well. Acute stress in rats leads to an increase in the hippocampal levels of H3K9me3, which, in turn, has a central role in the transcriptional repression of TEs, as already described above in terms of its implication in the control of their expression during early embryonic development and the consequent role in transcriptional plasticity of neural circuitry [231].

As a general note, it is worth mentioning that attempts to comprehensively evaluate the TE contribution to the enhancer landscape associated with mammalian-specific brain features, such as the neocortex, are revealing complex scenarios in which TE exaptation, when recognizable, contributes only in part to the whole enhancer landscape [232]. Such scenarios also include poorly characterized, interspersed repeats of uncertain classification, such as the MER130 repeat family, which has been shown to provide key TF binding sites to mouse neocortex developmental enhancers at a specific stage of embryo development [233].

### 5.2. HERV-Mediated Shaping of Genic and Transcriptional Patterns in the Human Brain

In line with the findings reported for mice, the primate-restricted KRAB zinc finger proteins—known for their role in silencing TE-embedded regulatory sequences during early embryogenesis—were also reported to control TE expression in the subsequent phases of development, as well as in adult tissues, leading to their co-optation in the genesis of brain transcription networks [234]. Particularly, ZNF417 and ZNF587 were shown on the one side to repress HERV-K(HML2) elements in human embryonic stem cells, and on the other side to control their expression in the developing and adult brain, influencing the differentiation and neurotransmission profile of neurons [234]. Of note, as already mentioned, the HERV-K(HML2) group is the most recently acquired group by primates, even showing polymorphic integrations in humans. Hence, in addition to their main role in human neuronal differentiation and physiology, these evolutionarily recent HERV transcriptional partners might account for variations in brain development and function in the human population. In relation to to the group’s recent acquisition, it has also recently been shown that a member of the HERV-K(HML2) group is still capable of producing an Env protein, expressed at a high level on the surface of pluripotent cells and involved in signaling pathways that regulate stem cell function [235]. Notably, the downregulation of this Env led to the dissociation of the stem cell colonies and prompted their differentiation along neuronal pathways, up to the production of neurons, suggesting a role in the regulation of embryonic neurodevelopment [235].

Given that HERVs are at the interface between self and non-self—being retroviral sequences endogenized by the host genome—another possible interaction with human physiology involves their residual ability to trigger innate immunity. This interaction is complex and multifaceted, possibly accounting either for pathogenesis or beneficial effects, as in the case of viral infections. In fact, in the presence of an exogenous virus, HERV expression has the potential to either worsen the clinical condition through cooperative effects or boost antiviral responses as a defense mechanism [60]. In this regard, as mentioned, the dispersion of HERVs in the human genome has been responsible for the shaping of pivotal antiviral networks. Particularly, LTRs of the MER41 primate-specific group were often found in the promoter regions of immune genes and were shown to serve as IFNγ-inducible enhancers [118]. Starting from this study and from the evidence that social behavior and neuronal connectivity in rodents have been shaped by the pro-inflammatory cytokine IFNγ, a similar interplay has been proposed for primate cognitive development, suggesting that—in parallel to the evolution of immune genes—the stepped self-domestication of MER41 could have contributed to key cognitive specificities found in hominins, including human language [236]. Accordingly, the promoter regions of human genes associated with intellectual disability are significantly enriched in MER41 LTR sequences, which hold binding sites for IFN-related transcription factors, such as STAT, YY1 and NFKB. Moreover, the localization of MER41 LTRs in the promoter regions of these genes is substantially different between humans and chimpanzees, possibly indicating a role of the group in cognitive changes that occurred after these species’ evolutionary split [236].

Another remarkable example of HERV-mediated shaping of brain gene expression is represented by a human-specific ERV insert shown to act as a tissue-specific enhancer for the schizophrenia-associated *PRODH* gene, coding for a proline dehydrogenase that is likely involved in neuromodulator synthesis in the central nervous system. The activity of this ERV-derived enhancer was shown to be regulated by DNA methylation in the hippocampus and to involve the binding of the SOX2 transcription factor [237].

In addition to the dispersion of *cis*-regulatory sites, the presence of HERV integrations also led to genic rearrangements during primate evolution. For example, *RHOXF2* is part of the homeobox genes, a family of key developmental regulators that are generally highly conserved. Despite this, *RHOXF2* showed an uncommon rapid evolution, with parallel gene duplications/losses in multiple primate lineages (especially during the origins of humans and chimpanzees) that had likely been mediated by the presence of ERV sequences flanking the gene, responsible for non-allelic recombination mechanism that in humans led to the presence of two *RHOXF2* copies [238]. Apart from its major function in primate spermatogenesis, *RHOXF2* may also be involved in brain functioning, probably through its direct regulation of three downstream genes (namely *UNC5C*, *PLTP* and *GDAP1*) that play important roles in the central nervous system. Interestingly, although both gene copies are equally expressed in the embryonic and newborn brain as well as in the adult testis, only one is still active in the adult brain, suggesting a possible role in central nervous system development [238]. In this scenario, given the dual functions of *RHOXF2* in the testis and brain, the observed between-copy gene expression divergence may represent a molecular mechanism that evolved to balance the potential functional conflicts between reproduction and cognition.

### 5.3. Old but Gold: Domestication of Ancient LTR Retrotransposons for Brain Physiology

Intriguingly, in addition to relatively young ERVs, the most ancient or defective LTR-retrotransposons can also still have a role in the host physiology, as reported for various Ty3/Gypsy retrotransposons. For example, in mice, the Mart family is formed by mammalian retrotransposons that are likely derived from a vertebrate Ty3/Gypsy family called Sushi, which in mammals has lost some structural features such as LTRs and the *pol* region, retaining a protein-coding region derived from the ancestral *gag* gene. In addition to their involvement in murine placenta development, some Mart-derived proteins have been proposed to serve as transcription factors regulating the expression of the myelin basic protein gene. Their potential involvement in brain functions is further suggested by the fact that disruption of the Mart4 gene in mice leads to abnormal cognitive behaviors, possibly involving the noradrenergic system [239]. Similarly, the SCAN gene family encodes for zinc-finger TFs having an *N*-terminal domain conserved in vertebrates and showing structural homologies to the C-terminal domain of retroviral capsids, probably derived from the domestication of a Gmr1-like LTR retrotransposon in an early tetrapod ancestor (about 300 million years ago) [239]. This ancient integration has thus been subject to subsequent duplication events, leading to about 70 and 40 related genes in humans and mice, respectively. The encoded TFs are involved in various biological processes, including the regulation of hippocampal cholesterol biosynthesis by the SCAN domain-containing NRIF, serving as a mediator of neuronal apoptosis and also interacting with the neurotrophin receptor p75 [240,241].

A further well-known example of a neurophysiologically relevant protein originating from the gag gene of an ancient Ty3/gypsy retrotransposon is the activity-regulated cytoskeleton-associated protein (ARC). The *ARC* gene was acquired before the divergence between mammals and amphibians and was repurposed during brain evolution to mediate communications between neural cells, having an important role in the development and plasticity of the nervous system [242]. The neuron-specific ARC protein exerts its role in mammalian excitatory synapses and is required for learning and memory processes [241]. Strikingly, the ARC protein has been shown to assemble into capsids that include mRNA sequences to be transferred from a neuron to new recipient cells through extracellular vesicles, then undergoing activity-dependent translation [243]. Homolog *Drosophila* proteins independently derived from the same family of retrotransposons and mediating similar processes at the neuromuscular junction provide an impressive example of convergent evolution, thus suggesting a deep entwining of ERV-derived proteins with nervous system evolution in animals [12].

Overall, as summarized in Table 1, various independent studies support an active role of evolutionarily fixed ERV integrations in the development and physiology of the mammalian brain, both through domestication of individual ERV loci to serve important coding functions and through the establishment of entire ERV-based transcriptional networks. In the case of humans, we anticipate that such an involvement will be further emphasized in the intensive studies of the possible association of HERV dysregulation with neurological and cognitive disorders.

## 6. Conclusions

**Figure 3:**
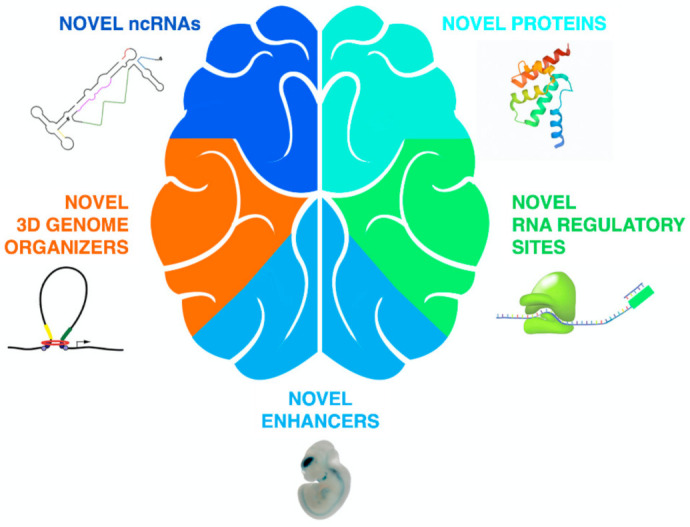
Summary of retrotransposon exaptations involved in brain evolution. Schematically recapitulated in the Figure are five major types of genomic novelty provided by RE exaptation that have been recognized to strongly affect brain evolution. Going clockwise from the left upper sector: REs can be a source of novel retrotransposon-derived ncRNAs (e.g., SINE-derived BC200 RNA); REs can be the source of novel proteins derived from retrotransposon (especially ERV) coding sequences; exonization of retrotransposon sequences (resulting in mRNAs or lncRNAs with embedded RE-derived sequences) can lead, for example, to the appearance of a new regulatory site (green box) within mRNA sequences, which can recruit miRNA, thereby affecting mRNA translation/stability; REs can provide new transcription TF binding sites, contributing to the birth of novel enhancers and altering the transcription of specific brain genes; REs can play a major role as 3D genome organizers, thereby strongly influencing the expression of brain-specific genes via changes in the 3D nuclear chromatin folding.

As detailed in the previous two sections, and summarized in Figure 3, both LTR and non-LTR retrotransposons appear to have contributed to mammalian brain evolution by acting as sources of novel ncRNAs, proteins, enhancers, RNA regulatory sites and sites for 3D genome organization.

To what extent, and according to which species-specific paths, brain functionality in mammals relies on retrotransposon exaptation will become increasingly clear as the particularly challenging aspects of retrotransposon molecular biology become easier to address. Major challenges include: (i) the unambiguous identification of expressed retrotransposon loci, together with the related issue of insertional and internal sequence polymorphisms [192], which becomes highly relevant when studies of retrotransposon impact are to be conducted at the population level; (ii) the implementation of a single-cell perspective while addressing the expression and genomic impact of retrotransposons, which appears particularly relevant in the case of brain biology given the extremely diverse specialization of neurons [193,244]; (iii) the experimental assessment of *cis*-regulatory roles of retrotransposons by means of their systematic perturbation, e.g., through CRISPR-based approaches [120].

Among the advances we expect in the nearest future, those relating to the role of retrotransposons in developmental gene control seem particularly relevant to the issue of the molecular mechanisms of brain evolution. Brain phenotypic differences between different mammalian species are likely to arise during brain development, and retrotransposons have recently taken center stage in the control of mammalian embryonic development, including neuronal differentiation [13,71,234]. Potentially relevant to this issue is the recent observation that the product of the *ADNP* gene, a transcriptional regulator involved in neuronal lineage development and associated with neurodevelopmental disorders [245], has the potential to recruit TFIIIC to a subset of Alu elements, thereby influencing their genome organization properties [141].

Finally, it is worth noting once more that retrotransposon-dependent genomic innovation, having contributed to human brain evolution and possibly underlying higher cognitive function, might also result in deleterious retrotransposon-dependent events, contributing to neurodevelopmental, neuropsychiatric and neurodegenerative diseases [18,183,246]. In this respect, future investigations systematically addressing the contribution of retrotransposons to the molecular changes underlying brain evolutionary transitions are likely to provide valuable new hints about complex neurological and neurodevelopmental disorders.

## Figures and Tables

**Table 1:** Main evidence concerning HERV loci and protein involvement in human brain development and physiology.

Name	Classification *	Retroviral Portion	Role in the Brain	Reference
multiple loci	HERV-K(HML2) [class II]	PBS	involved in neuronal differentiation and neurotransmission profile	[234]
multiple loci	MER41 [class I]	LTRs	serve as IFNγ-inducible enhancers in the promoter regions of immune genes, linked to human-specific cognitive functions	[236]
Xq24	HERV-I [class I]	whole provirus	flanking integrations mediating non-allelic recombination of *RHOXF2* gene copies, with a possible role in central nervous system development	[238]
NRIF	Gypsy/Ty3-like retrotransposons	Gag protein (capsid)	derived from domestication of a Gmr1-like LTR retrotransposon, mediates neuronal apoptosis and interacts with the neurotrophin receptor p75	[240]
Arc	Gypsy/Ty3-like retrotransposons	Gag protein (capsid)	mediate communications between neural cells, involved in development and plasticity of the nervous system	[243]

* Based on the classification from [58].

## References

[1] Burns K.H., Boeke J.D. (2012). Human transposon tectonics. Cell.

[2] Brosius J. (2019). Exaptation at the molecular genetic level. Sci. China Life Sci..

[3] Warren I.A., Naville M., Chalopin D., Levin P., Berger C.S., Galiana D., Volff J.N. (2015). Evolutionary impact of transposable elements on genomic diversity and lineage-specific innovation in vertebrates. Chromosome Res..

[4] Wells J.N., Feschotte C. (2020). A Field Guide to Eukaryotic Transposable Elements. Annu. Rev. Genet..

[5] Choi J.Y., Lee Y.C.G. (2020). Double-edged sword: The evolutionary consequences of the epigenetic silencing of transposable elements. PLoS Genet..

[6] Rebollo R., Romanish M.T., Mager D.L. (2012). Transposable elements: An abundant and natural source of regulatory sequences for host genes. Annu. Rev. Genet..

[7] Brosius J. (2014). The persistent contributions of RNA to eukaryotic gen(om)e architecture and cellular function. Cold Spring Harb. Perspect. Biol..

[8] Shapiro J.A. (2017). Exploring the read-write genome: Mobile DNA and mammalian adaptation. Crit. Rev. Biochem. Mol. Biol..

[9] Paquola A.C.M., Erwin J.A., Gage F.H. (2017). Insights into the role of somatic mosaicism in the brain. Curr. Opin. Syst. Biol..

[10] Bodea G.O., McKelvey E.G.Z., Faulkner G.J. (2018). Retrotransposon-induced mosaicism in the neural Genome. Open. Biol..

[11] Evans T.A., Erwin J.A. (2020). Retroelement-derived RNA and its role in the brain. Semin. Cell. Dev. Biol..

[12] Hantak M.P., Einstein J., Kearns R.B., Shepherd J.D. (2021). Intercellular Communication in the Nervous System Goes Viral. Trends Neurosci..

[13] Nishihara H. (2020). Transposable elements as genetic accelerators of evolution: Contribution to genome size, gene regulatory network rewiring and morphological innovation. Genes Genet. Syst..

[14] Luo X., Liu Y., Dang D., Hu T., Hou Y., Meng X., Zhang F., Li T., Wang C., Li M. (2021). 3D Genome of macaque fetal brain reveals evolutionary innovations during primate corticogenesis. Cell.

[15] Ahmadi A., De Toma I., Vilor-Tejedor N., Eftekhariyan Ghamsari M.R., Sadeghi I. (2020). Transposable elements in brain health and disease. Ageing Res. Rev..

[16] Reilly M.T., Faulkner G.J., Dubnau J., Ponomarev I., Gage F.H. (2013). The role of transposable elements in health and diseases of the central nervous system. J. Neurosci..

[17] Bitar M., Barry G. (2018). Multiple Innovations in Genetic and Epigenetic Mechanisms Cooperate to Underpin Human Brain Evolution. Mol. Biol. Evol..

[18] Larsen P.A., Hunnicutt K.E., Larsen R.J., Yoder A.D., Saunders A.M. (2018). Warning SINEs: Alu elements, evolution of the human brain, and the spectrum of neurological disease. Chromosome Res..

[19] Bourque G., Burns K.H., Gehring M., Gorbunova V., Seluanov A., Hammell M., Imbeault M., Izsvak Z., Levin H.L., Macfarlan T.S. (2018). Ten things you should know about transposable elements. Genome Biol..

[20] Fedoroff N.V. (2012). Transposable elements, epigenetics, and genome evolution. Science.

[21] McClintock B. (1984). The significance of responses of the genome to challenge. Science.

[22] Davidson E.H., Britten R.J. (1979). Regulation of gene expression: Possible role of repetitive sequences. Science.

[23] Biemont C. (2010). A brief history of the status of transposable elements: From junk DNA to major players in evolution. Genetics.

[24] Kazazian H.H., Moran J.V. (2017). Mobile DNA in Health and Disease. N. Engl. J. Med..

[25] Hancks D.C., Kazazian H.H. (2016). Roles for retrotransposon insertions in human disease. Mob. DNA.

[26] Levin H.L., Moran J.V. (2011). Dynamic interactions between transposable elements and their hosts. Nat. Rev. Genet..

[27] Chuong E.B., Rumi M.A., Soares M.J., Baker J.C. (2013). Endogenous retroviruses function as species-specific enhancer elements in the placenta. Nat. Genet..

[28] Bruno M., Mahgoub M., Macfarlan T.S. (2019). The Arms Race Between KRAB-Zinc Finger Proteins and Endogenous Retroelements and Its Impact on Mammals. Annu. Rev. Genet..

[29] Kojima K.K. (2018). Human transposable elements in Repbase: Genomic footprints from fish to humans. Mob. DNA.

[30] Storer J., Hubley R., Rosen J., Wheeler T.J., Smit A.F. (2021). The Dfam community resource of transposable element families, sequence models, and genome annotations. Mob. DNA.

[31] Kajikawa M., Okada N. (2002). LINEs mobilize SINEs in the eel through a shared 3’ sequence. Cell.

[32] Dewannieux M., Esnault C., Heidmann T. (2003). LINE-mediated retrotransposition of marked Alu sequences. Nat. Genet..

[33] Dieci G., Conti A., Pagano A., Carnevali D. (2013). Identification of RNA polymerase III-transcribed genes in eukaryotic genomes. Biochim. Biophys. Acta.

[34] Hermant C., Torres-Padilla M.E. (2021). TFs for TEs: The transcription factor repertoire of mammalian transposable elements. Genes Dev..

[35] Orioli A., Pascali C., Pagano A., Teichmann M., Dieci G. (2012). RNA polymerase III transcription control elements: Themes and variations. Gene.

[36] Sotero-Caio C.G., Platt R.N., Suh A., Ray D.A. (2017). Evolution and Diversity of Transposable Elements in Vertebrate Genomes. Genome Biol. Evol..

[37] Richardson S.R., Doucet A.J., Kopera H.C., Moldovan J.B., Garcia-Perez J.L., Moran J.V. (2015). The Influence of LINE-1 and SINE Retrotransposons on Mammalian Genomes. Microbiol. Spectr..

[38] Platt R.N., Vandewege M.W., Ray D.A. (2018). Mammalian transposable elements and their impacts on genome evolution. Chromosome Res..

[39] Blumenstiel J.P. (2019). Birth, School, Work, Death, and Resurrection: The Life Stages and Dynamics of Transposable Element Proliferation. Genes.

[40] Hormozdiari F., Konkel M.K., Prado-Martinez J., Chiatante G., Herraez I.H., Walker J.A., Nelson B., Alkan C., Sudmant P.H., Huddleston J. (2013). Rates and patterns of great ape retrotransposition. Proc. Natl. Acad. Sci. USA.

[41] Wang H., Xing J., Grover D., Hedges D.J., Han K., Walker J.A., Batzer M.A. (2005). SVA elements: A hominid-specific retroposon family. J. Mol. Biol..

[42] Hancks D.C., Kazazian H.H. (2010). SVA retrotransposons: Evolution and genetic instability. Semin. Cancer Biol..

[43] Maquat L.E. (2020). Short interspersed nuclear element (SINE)-mediated post-transcriptional effects on human and mouse gene expression: SINE-UP for active duty. Philos. Trans. R. Soc. Lond. B Biol. Sci..

[44] Kramerov D.A., Vassetzky N.S. (2011). SINEs. Wiley. Interdiscip. Rev. RNA.

[45] Jurka J., Bao W., Kojima K.K. (2011). Families of transposable elements, population structure and the origin of species. Biol. Direct..

[46] Jurka J., Zietkiewicz E., Labuda D. (1995). Ubiquitous mammalian-wide interspersed repeats (MIRs) are molecular fossils from the mesozoic era. Nucleic Acids Res..

[47] Smit A.F., Riggs A.D. (1995). MIRs are classic, tRNA-derived SINEs that amplified before the mammalian radiation. Nucleic Acids Res..

[48] Carnevali D., Conti A., Pellegrini M., Dieci G. (2017). Whole-genome expression analysis of mammalian-wide interspersed repeat elements in human cell lines. DNA Res. Int. J. Rapid. Publ. Rep. Genes Genomes.

[49] Greenwood A.D., Ishida Y., O’Brien S.P., Roca A.L., Eiden M.V. (2018). Transmission, Evolution, and Endogenization: Lessons Learned from Recent Retroviral Invasions. Microbiol. Mol. Biol. Rev..

[50] Dewannieux M., Heidmann T. (2013). Endogenous retroviruses: Acquisition, amplification and taming of genome invaders. Curr. Opin. Virol..

[51] Hayward A., Cornwallis C.K., Jern P. (2015). Pan-vertebrate comparative genomics unmasks retrovirus macroevolution. Proc. Natl. Acad. Sci. USA.

[52] Johnson W.E. (2019). Origins and evolutionary consequences of ancient endogenous retroviruses. Nat. Rev. Microbiol..

[53] Yohn C.T., Jiang Z., McGrath S.D., Hayden K.E., Khaitovich P., Johnson M.E., Eichler M.Y., McPherson J.D., Zhao S., Paabo S. (2005). Lineage-specific expansions of retroviral insertions within the genomes of African great apes but not humans and orangutans. PLoS Biol..

[54] Cordaux R., Batzer M.A. (2009). The impact of retrotransposons on human genome evolution. Nat. Rev. Genet..

[55] Grandi N., Tramontano E. (2018). HERV Envelope Proteins: Physiological Role and Pathogenic Potential in Cancer and Autoimmunity. Front. Microbiol..

[56] Grandi N., Tramontano E. (2017). Type W Human Endogenous Retrovirus (HERV-W) Integrations and Their Mobilization by L1 Machinery: Contribution to the Human Transcriptome and Impact on the Host Physiopathology. Viruses.

[57] Liu C.H., Grandi N., Palanivelu L., Tramontano E., Lin L.T. (2020). Contribution of Human Retroviruses to Disease Development-A Focus on the HIV- and HERV-Cancer Relationships and Treatment Strategies. Viruses.

[58] Vargiu L., Rodriguez-Tome P., Sperber G.O., Cadeddu M., Grandi N., Blikstad V., Tramontano E., Blomberg J. (2016). Classification and characterization of human endogenous retroviruses; mosaic forms are common. Retrovirology.

[59] Grandi N., Cadeddu M., Blomberg J., Tramontano E. (2016). Contribution of type W human endogenous retroviruses to the human genome: Characterization of HERV-W proviral insertions and processed pseudogenes. Retrovirology.

[60] Grandi N., Cadeddu M., Pisano M.P., Esposito F., Blomberg J., Tramontano E. (2017). Identification of a novel HERV-K(HML10): Comprehensive characterization and comparative analysis in non-human primates provide insights about HML10 proviruses structure and diffusion. Mob. DNA.

[61] Pisano M.P., Grandi N., Cadeddu M., Blomberg J., Tramontano E. (2019). Comprehensive Characterization of the Human Endogenous Retrovirus HERV-K(HML-6) Group: Overview of Structure, Phylogeny, and Contribution to the Human Genome. J. Virol..

[62] Subramanian R.P., Wildschutte J.H., Russo C., Coffin J.M. (2011). Identification, characterization, and comparative genomic distribution of the HERV-K (HML-2) group of human endogenous retroviruses. Retrovirology.

[63] Pisano M.P., Grandi N., Tramontano E. (2020). High-Throughput Sequencing is a Crucial Tool to Investigate the Contribution of Human Endogenous Retroviruses (HERVs) to Human Biology and Development. Viruses.

[64] Brosius J. (1991). Retroposons--seeds of evolution. Science.

[65] Jurka J. (1998). Repeats in genomic DNA: Mining and meaning. Curr. Opin. Struct. Biol..

[66] Deininger P.L., Moran J.V., Batzer M.A., Kazazian H.H. (2003). Mobile elements and mammalian genome evolution. Curr. Opin. Genet. Dev..

[67] Kazazian H.H. (2004). Mobile elements: Drivers of genome evolution. Science.

[68] Goodier J.L., Kazazian H.H. (2008). Retrotransposons revisited: The restraint and rehabilitation of parasites. Cell.

[69] Okada N., Sasaki T., Shimogori T., Nishihara H. (2010). Emergence of mammals by emergency: Exaptation. Genes Cells.

[70] Gifford W.D., Pfaff S.L., Macfarlan T.S. (2013). Transposable elements as genetic regulatory substrates in early development. Trends Cell. Biol..

[71] Friedli M., Trono D. (2015). The Developmental Control of Transposable Elements and the Evolution of Higher Species. Annu. Rev. Cell. Dev. Biol..

[72] Garcia-Perez J.L., Widmann T.J., Adams I.R. (2016). The impact of transposable elements on mammalian development. Development.

[73] Cosby R.L., Chang N.C., Feschotte C. (2019). Host-transposon interactions: Conflict, cooperation, and cooption. Genes Dev..

[74] Mita P., Boeke J.D. (2016). How retrotransposons shape genome regulation. Curr. Opin. Genet. Dev..

[75] Etchegaray E., Naville M., Volff J.N., Haftek-Terreau Z. (2021). Transposable element-derived sequences in vertebrate development. Mob. DNA.

[76] Chuong E.B., Elde N.C., Feschotte C. (2017). Regulatory activities of transposable elements: From conflicts to benefits. Nat. Rev. Genet..

[77] Elbarbary R.A., Lucas B.A., Maquat L.E. (2016). Retrotransposons as regulators of gene expression. Science.

[78] Thompson P.J., Macfarlan T.S., Lorincz M.C. (2016). Long Terminal Repeats: From Parasitic Elements to Building Blocks of the Transcriptional Regulatory Repertoire. Mol. Cell..

[79] Chen S., Krinsky B.H., Long M. (2013). New genes as drivers of phenotypic evolution. Nat. Rev. Genet..

[80] Cheetham S.W., Faulkner G.J., Dinger M.E. (2020). Overcoming challenges and dogmas to understand the functions of pseudogenes. Nat. Rev. Genet..

[81] Carmi S., Church G.M., Levanon E.Y. (2011). Large-scale DNA editing of retrotransposons accelerates mammalian genome evolution. Nat. Commun..

[82] Daniel C., Silberberg G., Behm M., Ohman M. (2014). Alu elements shape the primate transcriptome by cis-regulation of RNA editing. Genome Biol..

[83] Sundaram V., Wysocka J. (2020). Transposable elements as a potent source of diverse cis-regulatory sequences in mammalian genomes. Philos. Trans. R. Soc. Lond. B Biol. Sci..

[84] Drongitis D., Aniello F., Fucci L., Donizetti A. (2019). Roles of Transposable Elements in the Different Layers of Gene Expression Regulation. Int. J. Mol. Sci..

[85] Lin L., Jiang P., Park J.W., Wang J., Lu Z.X., Lam M.P., Ping P., Xing Y. (2016). The contribution of Alu exons to the human proteome. Genome Biol..

[86] Kim E.Z., Wespiser A.R., Caffrey D.R. (2016). The domain structure and distribution of Alu elements in long noncoding RNAs and mRNAs. RNA.

[87] Toki N., Takahashi H., Sharma H., Valentine M.N.Z., Rahman F.M., Zucchelli S., Gustincich S., Carninci P. (2020). SINEUP long non-coding RNA acts via PTBP1 and HNRNPK to promote translational initiation assemblies. Nucleic Acids Res..

[88] Chen L.L., Yang L. (2017). ALUternative Regulation for Gene Expression. Trends Cell. Biol..

[89] Damert A., Raiz J., Horn A.V., Lower J., Wang H., Xing J., Batzer M.A., Lower R., Schumann G.G. (2009). 5’-Transducing SVA retrotransposon groups spread efficiently throughout the human Genome. Genome Res..

[90] Wu Y., Zhao W., Liu Y., Tan X., Li X., Zou Q., Xiao Z., Xu H., Wang Y., Yang X. (2018). Function of HNRNPC in breast cancer cells by controlling the dsRNA-induced interferon response. EMBO J..

[91] Smalheiser N.R., Torvik V.I. (2006). Alu elements within human mRNAs are probable microRNA targets. Trends Genet..

[92] Gong C., Maquat L.E. (2011). lncRNAs transactivate STAU1-mediated mRNA decay by duplexing with 3′ UTRs via Alu elements. Nature.

[93] Welden J.R., Stamm S. (2019). Pre-mRNA structures forming circular RNAs. Biochim. Biophys. Acta Gene Regul. Mech..

[94] Patop I.L., Wust S., Kadener S. (2019). Past, present, and future of circRNAs. EMBO J..

[95] Di Timoteo G., Rossi F., Bozzoni I. (2020). Circular RNAs in cell differentiation and development. Development.

[96] Lee H.E., Huh J.W., Kim H.S. (2020). Bioinformatics Analysis of Evolution and Human Disease Related Transposable Element-Derived microRNAs. Life.

[97] Smalheiser N.R., Torvik V.I. (2005). Mammalian microRNAs derived from genomic repeats. Trends Genet..

[98] Piriyapongsa J., Marino-Ramirez L., Jordan I.K. (2007). Origin and evolution of human microRNAs from transposable elements. Genetics.

[99] Roberts J.T., Cooper E.A., Favreau C.J., Howell J.S., Lane L.G., Mills J.E., Newman D.C., Perry T.J., Russell M.E., Wallace B.M. (2013). Continuing analysis of microRNA origins: Formation from transposable element insertions and noncoding RNA mutations. Mob. Genet. Elem..

[100] Spengler R.M., Oakley C.K., Davidson B.L. (2014). Functional microRNAs and target sites are created by lineage-specific transposition. Hum. Mol. Genet..

[101] Lavialle C., Cornelis G., Dupressoir A., Esnault C., Heidmann O., Vernochet C., Heidmann T. (2013). Paleovirology of ‘syncytins’, retroviral env genes exapted for a role in placentation. Philos. Trans. R. Soc. Lond. B Biol. Sci..

[102] Blaise S., de Parseval N., Benit L., Heidmann T. (2003). Genomewide screening for fusogenic human endogenous retrovirus envelopes identifies syncytin 2, a gene conserved on primate evolution. Proc. Natl. Acad. Sci. USA.

[103] Lokossou A.G., Toudic C., Barbeau B. (2014). Implication of human endogenous retrovirus envelope proteins in placental functions. Viruses.

[104] Long H.K., Prescott S.L., Wysocka J. (2016). Ever-Changing Landscapes: Transcriptional Enhancers in Development and Evolution. Cell.

[105] Wagner G.P., Lynch V.J. (2010). Evolutionary novelties. Curr. Biol..

[106] Sundaram V., Cheng Y., Ma Z., Li D., Xing X., Edge P., Snyder M.P., Wang T. (2014). Widespread contribution of transposable elements to the innovation of gene regulatory networks. Genome Res..

[107] Marnetto D., Mantica F., Molineris I., Grassi E., Pesando I., Provero P. (2018). Evolutionary Rewiring of Human Regulatory Networks by Waves of Genome Expansion. Am. J. Hum. Genet..

[108] Lynch V.J., Nnamani M.C., Kapusta A., Brayer K., Plaza S.L., Mazur E.C., Emera D., Sheikh S.Z., Grutzner F., Bauersachs S. (2015). Ancient transposable elements transformed the uterine regulatory landscape and transcriptome during the evolution of mammalian pregnancy. Cell Rep..

[109] Imbeault M., Helleboid P.Y., Trono D. (2017). KRAB zinc-finger proteins contribute to the evolution of gene regulatory networks. Nature.

[110] Pontis J., Planet E., Offner S., Turelli P., Duc J., Coudray A., Theunissen T.W., Jaenisch R., Trono D. (2019). Hominoid-Specific Transposable Elements and KZFPs Facilitate Human Embryonic Genome Activation and Control Transcription in Naive Human ESCs. Cell Stem Cell.

[111] Chuong E.B. (2013). Retroviruses facilitate the rapid evolution of the mammalian placenta. Bioessays.

[112] Sundaram V., Choudhary M.N., Pehrsson E., Xing X., Fiore C., Pandey M., Maricque B., Udawatta M., Ngo D., Chen Y. (2017). Functional cis-regulatory modules encoded by mouse-specific endogenous retrovirus. Nat. Commun..

[113] Field A., Adelman K. (2020). Evaluating Enhancer Function and Transcription. Annu. Rev. Biochem..

[114] Ernst J., Kellis M. (2017). Chromatin-state discovery and genome annotation with ChromHMM. Nat. Protoc..

[115] Pehrsson E.C., Choudhary M.N.K., Sundaram V., Wang T. (2019). The epigenomic landscape of transposable elements across normal human development and anatomy. Nat. Commun..

[116] Jacques P.E., Jeyakani J., Bourque G. (2013). The majority of primate-specific regulatory sequences are derived from transposable elements. PLoS Genet..

[117] Trizzino M., Park Y., Holsbach-Beltrame M., Aracena K., Mika K., Caliskan M., Perry G.H., Lynch V.J., Brown C.D. (2017). Transposable elements are the primary source of novelty in primate gene regulation. Genome Res..

[118] Chuong E.B., Elde N.C., Feschotte C. (2016). Regulatory evolution of innate immunity through co-option of endogenous retroviruses. Science.

[119] Wang T., Zeng J., Lowe C.B., Sellers R.G., Salama S.R., Yang M., Burgess S.M., Brachmann R.K., Haussler D. (2007). Species-specific endogenous retroviruses shape the transcriptional network of the human tumor suppressor protein p53. Proc. Natl. Acad. Sci. USA.

[120] Fuentes D.R., Swigut T., Wysocka J. (2018). Systematic perturbation of retroviral LTRs reveals widespread long-range effects on human gene regulation. Elife.

[121] Prescott S.L., Srinivasan R., Marchetto M.C., Grishina I., Narvaiza I., Selleri L., Gage F.H., Swigut T., Wysocka J. (2015). Enhancer divergence and cis-regulatory evolution in the human and chimp neural crest. Cell.

[122] Su M., Han D., Boyd-Kirkup J., Yu X., Han J.D. (2014). Evolution of Alu elements toward enhancers. Cell Rep..

[123] Policarpi C., Crepaldi L., Brookes E., Nitarska J., French S.M., Coatti A., Riccio A. (2017). Enhancer SINEs Link Pol III to Pol II Transcription in Neurons. Cell Rep..

[124] Kempfer R., Pombo A. (2020). Methods for mapping 3D chromosome architecture. Nat. Rev. Genet..

[125] Szabo Q., Bantignies F., Cavalli G. (2019). Principles of genome folding into topologically associating domains. Sci. Adv..

[126] Lu J.Y., Chang L., Li T., Wang T., Yin Y., Zhan G., Han X., Zhang K., Tao Y., Percharde M. (2021). Homotypic clustering of L1 and B1/Alu repeats compartmentalizes the 3D Genome. Cell Res..

[127] Cavalheiro G.R., Pollex T., Furlong E.E. (2021). To loop or not to loop: What is the role of TADs in enhancer function and gene regulation?. Curr. Opin. Genet. Dev..

[128] Schoenfelder S., Fraser P. (2019). Long-range enhancer-promoter contacts in gene expression control. Nat. Rev. Genet..

[129] Rowley M.J., Corces V.G. (2018). Organizational principles of 3D genome architecture. Nat. Rev. Genet..

[130] Schmitt A.D., Hu M., Ren B. (2016). Genome-wide mapping and analysis of chromosome architecture. Nat. Rev. Mol. Cell. Biol..

[131] Bonev B., Cavalli G. (2016). Organization and function of the 3D Genome. Nat. Rev. Genet..

[132] Pombo A., Dillon N. (2015). Three-dimensional genome architecture: Players and mechanisms. Nat. Rev. Mol. Cell. Biol..

[133] Xiang J.F., Corces V.G. (2020). Regulation of 3D chromatin organization by CTCF. Curr. Opin. Genet. Dev..

[134] Ong C.T., Corces V.G. (2014). CTCF: An architectural protein bridging genome topology and function. Nat. Rev. Genet..

[135] Nora E.P., Lajoie B.R., Schulz E.G., Giorgetti L., Okamoto I., Servant N., Piolot T., van Berkum N.L., Meisig J., Sedat J. (2012). Spatial partitioning of the regulatory landscape of the X-inactivation centre. Nature.

[136] Kentepozidou E., Aitken S.J., Feig C., Stefflova K., Ibarra-Soria X., Odom D.T., Roller M., Flicek P. (2020). Clustered CTCF binding is an evolutionary mechanism to maintain topologically associating domains. Genome Biol..

[137] Kaaij L.J.T., Mohn F., van der Weide R.H., de Wit E., Buhler M. (2019). The ChAHP Complex Counteracts Chromatin Looping at CTCF Sites that Emerged from SINE Expansions in Mouse. Cell.

[138] Choudhary M.N., Friedman R.Z., Wang J.T., Jang H.S., Zhuo X., Wang T. (2020). Co-opted transposons help perpetuate conserved higher-order chromosomal structures. Genome Biol..

[139] Ichiyanagi T., Katoh H., Mori Y., Hirafuku K., Boyboy B.A., Kawase M., Ichiyanagi K. (2021). B2 SINE copies serve as a transposable boundary of DNA methylation and histone modifications in the mouse. Mol. Biol. Evol..

[140] Wang J., Vicente-Garcia C., Seruggia D., Molto E., Fernandez-Minan A., Neto A., Lee E., Gomez-Skarmeta J.L., Montoliu L., Lunyak V.V. (2015). MIR retrotransposon sequences provide insulators to the human Genome. Proc. Natl. Acad. Sci. USA.

[141] Ferrari R., de Llobet Cucalon L.I., Di Vona C., Le Dilly F., Vidal E., Lioutas A., Oliete J.Q., Jochem L., Cutts E., Dieci G. (2020). TFIIIC Binding to Alu Elements Controls Gene Expression via Chromatin Looping and Histone Acetylation. Mol. Cell.

[142] Crepaldi L., Policarpi C., Coatti A., Sherlock W.T., Jongbloets B.C., Down T.A., Riccio A. (2013). Binding of TFIIIC to sine elements controls the relocation of activity-dependent neuronal genes to transcription factories. PLoS Genet..

[143] Conti A., Carnevali D., Bollati V., Fustinoni S., Pellegrini M., Dieci G. (2015). Identification of RNA polymerase III-transcribed Alu loci by computational screening of RNA-Seq data. Nucleic Acids Res..

[144] Zhang X.O., Gingeras T.R., Weng Z. (2019). Genome-wide analysis of polymerase III-transcribed Alu elements suggests cell-type-specific enhancer function. Genome Res..

[145] Zhang Y., Li T., Preissl S., Amaral M.L., Grinstein J.D., Farah E.N., Destici E., Qiu Y., Hu R., Lee A.Y. (2019). Transcriptionally active HERV-H retrotransposons demarcate topologically associating domains in human pluripotent stem cells. Nat. Genet..

[146] Striedter G.F., Northcutt R.G. (2020). Brains through Time. A Natural History of Vertebrates.

[147] Briscoe S.D., Ragsdale C.W. (2018). Homology, neocortex, and the evolution of developmental mechanisms. Science.

[148] Paolino A., Fenlon L.R., Kozulin P., Haines E., Lim J.W.C., Richards L.J., Suarez R. (2020). Differential timing of a conserved transcriptional network underlies divergent cortical projection routes across mammalian brain evolution. Proc. Natl. Acad. Sci. USA.

[149] Olkowicz S., Kocourek M., Lucan R.K., Portes M., Fitch W.T., Herculano-Houzel S., Nemec P. (2016). Birds have primate-like numbers of neurons in the forebrain. Proc. Natl. Acad. Sci. USA.

[150] Briscoe S.D., Ragsdale C.W. (2019). Evolution of the Chordate Telencephalon. Curr. Biol..

[151] Kaas J.H. (2013). The evolution of brains from early mammals to humans. Wiley Interdiscip. Rev. Cogn. Sci..

[152] Fernandez V., Llinares-Benadero C., Borrell V. (2016). Cerebral cortex expansion and folding: What have we learned?. EMBO J..

[153] Krubitzer L.A., Prescott T.J. (2018). The Combinatorial Creature: Cortical Phenotypes within and across Lifetimes. Trends Neurosci..

[154] Sousa A.M.M., Meyer K.A., Santpere G., Gulden F.O., Sestan N. (2017). Evolution of the Human Nervous System Function, Structure, and Development. Cell.

[155] Suzuki I.K. (2020). Molecular drivers of human cerebral cortical evolution. Neurosci. Res..

[156] Enard W. (2016). The Molecular Basis of Human Brain Evolution. Curr. Biol..

[157] Fiddes I.T., Lodewijk G.A., Mooring M., Bosworth C.M., Ewing A.D., Mantalas G.L., Novak A.M., van den Bout A., Bishara A., Rosenkrantz J.L. (2018). Human-Specific NOTCH2NL Genes Affect Notch Signaling and Cortical Neurogenesis. Cell.

[158] Suzuki I.K., Gacquer D., Van Heurck R., Kumar D., Wojno M., Bilheu A., Herpoel A., Lambert N., Cheron J., Polleux F. (2018). Human-Specific NOTCH2NL Genes Expand Cortical Neurogenesis through Delta/Notch Regulation. Cell.

[159] Bizzotto S., Walsh C.A. (2018). Making a Notch in the Evolution of the Human Cortex. Dev. Cell.

[160] Dennis M.Y., Nuttle X., Sudmant P.H., Antonacci F., Graves T.A., Nefedov M., Rosenfeld J.A., Sajjadian S., Malig M., Kotkiewicz H. (2012). Evolution of human-specific neural SRGAP2 genes by incomplete segmental duplication. Cell.

[161] Schmidt E.R.E., Kupferman J.V., Stackmann M., Polleux F. (2019). The human-specific paralogs SRGAP2B and SRGAP2C differentially modulate SRGAP2A-dependent synaptic development. Sci. Rep..

[162] Charrier C., Joshi K., Coutinho-Budd J., Kim J.E., Lambert N., de Marchena J., Jin W.L., Vanderhaeghen P., Ghosh A., Sassa T. (2012). Inhibition of SRGAP2 function by its human-specific paralogs induces neoteny during spine maturation. Cell.

[163] Florio M., Albert M., Taverna E., Namba T., Brandl H., Lewitus E., Haffner C., Sykes A., Wong F.K., Peters J. (2015). Human-specific gene ARHGAP11B promotes basal progenitor amplification and neocortex expansion. Science.

[164] Heide M., Haffner C., Murayama A., Kurotaki Y., Shinohara H., Okano H., Sasaki E., Huttner W.B. (2020). Human-specific ARHGAP11B increases size and folding of primate neocortex in the fetal marmoset. Science.

[165] Namba T., Doczi J., Pinson A., Xing L., Kalebic N., Wilsch-Brauninger M., Long K.R., Vaid S., Lauer J., Bogdanova A. (2020). Human-Specific ARHGAP11B Acts in Mitochondria to Expand Neocortical Progenitors by Glutaminolysis. Neuron.

[166] Levchenko A., Kanapin A., Samsonova A., Gainetdinov R.R. (2018). Human Accelerated Regions and Other Human-Specific Sequence Variations in the Context of Evolution and Their Relevance for Brain Development. Genome Biol. Evol..

[167] Haygood R., Babbitt C.C., Fedrigo O., Wray G.A. (2010). Contrasts between adaptive coding and noncoding changes during human evolution. Proc. Natl. Acad. Sci. USA.

[168] Capra J.A., Erwin G.D., McKinsey G., Rubenstein J.L., Pollard K.S. (2013). Many human accelerated regions are developmental enhancers. Philos. Trans. R. Soc. Lond. B Biol. Sci..

[169] Gittelman R.M., Hun E., Ay F., Madeoy J., Pennacchio L., Noble W.S., Hawkins R.D., Akey J.M. (2015). Comprehensive identification and analysis of human accelerated regulatory DNA. Genome Res..

[170] Doan R.N., Bae B.I., Cubelos B., Chang C., Hossain A.A., Al-Saad S., Mukaddes N.M., Oner O., Al-Saffar M., Balkhy S. (2016). Mutations in Human Accelerated Regions Disrupt Cognition and Social Behavior. Cell.

[171] Kamm G.B., Pisciottano F., Kliger R., Franchini L.F. (2013). The developmental brain gene NPAS3 contains the largest number of accelerated regulatory sequences in the human Genome. Mol. Biol. Evol..

[172] Boyd J.L., Skove S.L., Rouanet J.P., Pilaz L.J., Bepler T., Gordan R., Wray G.A., Silver D.L. (2015). Human-chimpanzee differences in a FZD8 enhancer alter cell-cycle dynamics in the developing neocortex. Curr. Biol..

[173] Ataman B., Boulting G.L., Harmin D.A., Yang M.G., Baker-Salisbury M., Yap E.L., Malik A.N., Mei K., Rubin A.A., Spiegel I. (2016). Evolution of Osteocrin as an activity-regulated factor in the primate brain. Nature.

[174] de la Torre-Ubieta L., Stein J.L., Won H., Opland C.K., Liang D., Lu D., Geschwind D.H. (2018). The Dynamic Landscape of Open Chromatin during Human Cortical Neurogenesis. Cell.

[175] Harabula I., Pombo A. (2021). The dynamics of chromatin architecture in brain development and function. Curr. Opin. Genet. Dev..

[176] Rajarajan P., Gil S.E., Brennand K.J., Akbarian S. (2016). Spatial genome organization and cognition. Nat. Rev. Neurosci..

[177] Pollard K.S., Salama S.R., Lambert N., Lambot M.A., Coppens S., Pedersen J.S., Katzman S., King B., Onodera C., Siepel A. (2006). An RNA gene expressed during cortical development evolved rapidly in humans. Nature.

[178] Heimberg A.M., Sempere L.F., Moy V.N., Donoghue P.C., Peterson K.J. (2008). MicroRNAs and the advent of vertebrate morphological complexity. Proc. Natl. Acad. Sci. USA.

[179] Somel M., Liu X., Tang L., Yan Z., Hu H., Guo S., Jiang X., Zhang X., Xu G., Xie G. (2011). MicroRNA-driven developmental remodeling in the brain distinguishes humans from other primates. PLoS Biol..

[180] Prodromidou K., Matsas R. (2019). Species-Specific miRNAs in Human Brain Development and Disease. Front. Cell. Neurosci..

[181] Zimmer-Bensch G. (2019). Emerging Roles of Long Non-Coding RNAs as Drivers of Brain Evolution. Cells.

[182] Briggs J.A., Wolvetang E.J., Mattick J.S., Rinn J.L., Barry G. (2015). Mechanisms of Long Non-coding RNAs in Mammalian Nervous System Development, Plasticity, Disease, and Evolution. Neuron.

[183] Larsen P.A., Lutz M.W., Hunnicutt K.E., Mihovilovic M., Saunders A.M., Yoder A.D., Roses A.D. (2017). The Alu neurodegeneration hypothesis: A primate-specific mechanism for neuronal transcription noise, mitochondrial dysfunction, and manifestation of neurodegenerative disease. Alzheimers Dement..

[184] Brosius J. (1999). RNAs from all categories generate retrosequences that may be exapted as novel genes or regulatory elements. Gene.

[185] Tiedge H., Chen W., Brosius J. (1993). Primary Structure, Neural-Specific Expression, and Dendritic Location of Human Bc200 Rna. J. Neurosci..

[186] Sosinska P., Mikula-Pietrasik J., Ksiazek K. (2015). The double-edged sword of long non-coding RNA: The role of human brain-specific BC200 RNA in translational control, neurodegenerative diseases, and cancer. Mutat. Res. Rev. Mutat. Res..

[187] Smalheiser N.R. (2014). The RNA-centred view of the synapse: Non-coding RNAs and synaptic plasticity. Philos. Trans. R. Soc. Lond. B Biol. Sci..

[188] Castelnuovo M., Massone S., Tasso R., Fiorino G., Gatti M., Robello M., Gatta E., Berger A., Strub K., Florio T. (2010). An Alu-like RNA promotes cell differentiation and reduces malignancy of human neuroblastoma cells. FASEB J. Off. Publ. Fed. Am. Soc. Exp. Biol..

[189] Parrott A.M., Tsai M., Batchu P., Ryan K., Ozer H.L., Tian B., Mathews M.B. (2011). The evolution and expression of the snaR family of small non-coding RNAs. Nucleic Acids Res..

[190] Carnevali D., Dieci G. (2017). Identification of RNA Polymerase III-Transcribed SINEs at Single-Locus Resolution from RNA Sequencing Data. Noncoding RNA.

[191] Guffanti G., Bartlett A., Klengel T., Klengel C., Hunter R., Glinsky G., Macciardi F. (2018). Novel Bioinformatics Approach Identifies Transcriptional Profiles of Lineage-Specific Transposable Elements at Distinct Loci in the Human Dorsolateral Prefrontal Cortex. Mol. Biol. Evol..

[192] Lanciano S., Cristofari G. (2020). Measuring and interpreting transposable element expression. Nat. Rev. Genet..

[193] Linker S.B., Randolph-Moore L., Kottilil K., Qiu F., Jaeger B.N., Barron J., Gage F.H. (2020). Identification of bona fide B2 SINE retrotransposon transcription through single-nucleus RNA-seq of the mouse hippocampus. Genome Res..

[194] Li J.B., Church G.M. (2013). Deciphering the functions and regulation of brain-enriched A-to-I RNA editing. Nat. Neurosci..

[195] Linker S.B., Marchetto M.C., Narvaiza I., Denli A.M., Gage F.H. (2017). Examining non-LTR retrotransposons in the context of the evolving primate brain. BMC Biol..

[196] Bhattacharya A., Jha V., Singhal K., Fatima M., Singh D., Chaturvedi G., Dholakia D., Kutum R., Pandey R., Bakken T.E. (2021). Multiple Alu Exonization in 3’UTR of a Primate-Specific Isoform of CYP20A1 Creates a Potential miRNA Sponge. Genome Biol. Evol..

[197] Fasolo F., Patrucco L., Volpe M., Bon C., Peano C., Mignone F., Carninci P., Persichetti F., Santoro C., Zucchelli S. (2019). The RNA-binding protein ILF3 binds to transposable element sequences in SINEUP lncRNAs. FASEB J. Off. Publ. Fed. Am. Soc. Exp. Biol..

[198] Carrieri C., Cimatti L., Biagioli M., Beugnet A., Zucchelli S., Fedele S., Pesce E., Ferrer I., Collavin L., Santoro C. (2012). Long non-coding antisense RNA controls Uchl1 translation through an embedded SINEB2 repeat. Nature.

[199] Schein A., Zucchelli S., Kauppinen S., Gustincich S., Carninci P. (2016). Identification of antisense long noncoding RNAs that function as SINEUPs in human cells. Sci. Rep..

[200] Zhou Y.H., Zheng J.B., Gu X., Saunders G.F., Yung W.K. (2002). Novel PAX6 binding sites in the human genome and the role of repetitive elements in the evolution of gene regulation. Genome Res..

[201] Zhou Y., Zheng J.B., Gu X., Li W., Saunders G.F. (2000). A novel Pax-6 binding site in rodent B1 repetitive elements: Coevolution between developmental regulation and repeated elements?. Gene.

[202] Bejerano G., Lowe C.B., Ahituv N., King B., Siepel A., Salama S.R., Rubin E.M., Kent W.J., Haussler D. (2006). A distal enhancer and an ultraconserved exon are derived from a novel retroposon. Nature.

[203] Santangelo A.M., de Souza F.S., Franchini L.F., Bumaschny V.F., Low M.J., Rubinstein M. (2007). Ancient exaptation of a CORE-SINE retroposon into a highly conserved mammalian neuronal enhancer of the proopiomelanocortin gene. PLoS Genet..

[204] Sasaki T., Nishihara H., Hirakawa M., Fujimura K., Tanaka M., Kokubo N., Kimura-Yoshida C., Matsuo I., Sumiyama K., Saitou N. (2008). Possible involvement of SINEs in mammalian-specific brain formation. Proc. Natl. Acad. Sci. USA.

[205] Tashiro K., Teissier A., Kobayashi N., Nakanishi A., Sasaki T., Yan K., Tarabykin V., Vigier L., Sumiyama K., Hirakawa M. (2011). A mammalian conserved element derived from SINE displays enhancer properties recapitulating Satb2 expression in early-born callosal projection neurons. PLoS ONE.

[206] Nakanishi A., Kobayashi N., Suzuki-Hirano A., Nishihara H., Sasaki T., Hirakawa M., Sumiyama K., Shimogori T., Okada N. (2012). A SINE-derived element constitutes a unique modular enhancer for mammalian diencephalic Fgf8. PLoS ONE.

[207] Nishihara H., Kobayashi N., Kimura-Yoshida C., Yan K., Bormuth O., Ding Q., Nakanishi A., Sasaki T., Hirakawa M., Sumiyama K. (2016). Coordinately Co-opted Multiple Transposable Elements Constitute an Enhancer for wnt5a Expression in the Mammalian Secondary Palate. PLoS Genet..

[208] Franchini L.F., Lopez-Leal R., Nasif S., Beati P., Gelman D.M., Low M.J., de Souza F.J., Rubinstein M. (2011). Convergent evolution of two mammalian neuronal enhancers by sequential exaptation of unrelated retroposons. Proc. Natl. Acad. Sci. USA.

[209] Pickard B.S., Pieper A.A., Porteous D.J., Blackwood D.H., Muir W.J. (2006). The NPAS3 gene--emerging evidence for a role in psychiatric illness. Ann. Med..

[210] Kamm G.B., Lopez-Leal R., Lorenzo J.R., Franchini L.F. (2013). A fast-evolving human NPAS3 enhancer gained reporter expression in the developing forebrain of transgenic mice. Philos. Trans. R. Soc. Lond. B Biol. Sci..

[211] Bailey J.A., Eichler E.E. (2006). Primate segmental duplications: Crucibles of evolution, diversity and disease. Nat. Rev. Genet..

[212] Bailey J.A., Liu G., Eichler E.E. (2003). An Alu transposition model for the origin and expansion of human segmental duplications. Am. J. Hum. Genet..

[213] Sen S.K., Han K., Wang J., Lee J., Wang H., Callinan P.A., Dyer M., Cordaux R., Liang P., Batzer M.A. (2006). Human genomic deletions mediated by recombination between Alu elements. Am. J. Hum. Genet..

[214] Muotri A.R., Chu V.T., Marchetto M.C., Deng W., Moran J.V., Gage F.H. (2005). Somatic mosaicism in neuronal precursor cells mediated by L1 retrotransposition. Nature.

[215] Ostertag E.M., Kazazian H.H. (2005). Genetics: LINEs in mind. Nature.

[216] Coufal N.G., Garcia-Perez J.L., Peng G.E., Yeo G.W., Mu Y., Lovci M.T., Morell M., O’Shea K.S., Moran J.V., Gage F.H. (2009). L1 retrotransposition in human neural progenitor cells. Nature.

[217] Muotri A.R., Marchetto M.C., Coufal N.G., Oefner R., Yeo G., Nakashima K., Gage F.H. (2010). L1 retrotransposition in neurons is modulated by MeCP2. Nature.

[218] Coufal N.G., Garcia-Perez J.L., Peng G.E., Marchetto M.C., Muotri A.R., Mu Y., Carson C.T., Macia A., Moran J.V., Gage F.H. (2011). Ataxia telangiectasia mutated (ATM) modulates long interspersed element-1 (L1) retrotransposition in human neural stem cells. Proc. Natl. Acad. Sci. USA.

[219] Baillie J.K., Barnett M.W., Upton K.R., Gerhardt D.J., Richmond T.A., De Sapio F., Brennan P.M., Rizzu P., Smith S., Fell M. (2011). Somatic retrotransposition alters the genetic landscape of the human brain. Nature.

[220] Evrony G.D., Cai X., Lee E., Hills L.B., Elhosary P.C., Lehmann H.S., Parker J.J., Atabay K.D., Gilmore E.C., Poduri A. (2012). Single-neuron sequencing analysis of L1 retrotransposition and somatic mutation in the human brain. Cell.

[221] Jonsson M.E., Ludvik Brattas P., Gustafsson C., Petri R., Yudovich D., Pircs K., Verschuere S., Madsen S., Hansson J., Larsson J. (2019). Activation of neuronal genes via LINE-1 elements upon global DNA demethylation in human neural progenitors. Nat. Commun..

[222] Nishihara H. (2019). Retrotransposons spread potential cis-regulatory elements during mammary gland evolution. Nucleic Acids Res..

[223] Roller M., Stamper E., Villar D., Izuogu O., Martin F., Redmond A.M., Ramachanderan R., Harewood L., Odom D.T., Flicek P. (2021). LINE retrotransposons characterize mammalian tissue-specific and evolutionarily dynamic regulatory regions. Genome Biol..

[224] Christensen T. (2016). Human endogenous retroviruses in neurologic disease. APMIS.

[225] Kury P., Nath A., Creange A., Dolei A., Marche P., Gold J., Giovannoni G., Hartung H.P., Perron H. (2018). Human Endogenous Retroviruses in Neurological Diseases. Trends Mol. Med..

[226] Perron H., Lang A. (2010). The human endogenous retrovirus link between genes and environment in multiple sclerosis and in multifactorial diseases associating neuroinflammation. Clin. Rev. Allergy Immunol..

[227] Lee K.H., Horiuchi M., Itoh T., Greenhalgh D.G., Cho K. (2011). Cerebellum-specific and age-dependent expression of an endogenous retrovirus with intact coding potential. Retrovirology.

[228] Mortelmans K., Wang-Johanning F., Johanning G.L. (2016). The role of human endogenous retroviruses in brain development and function. APMIS.

[229] Rowe H.M., Jakobsson J., Mesnard D., Rougemont J., Reynard S., Aktas T., Maillard P.V., Layard-Liesching H., Verp S., Marquis J. (2010). KAP1 controls endogenous retroviruses in embryonic stem cells. Nature.

[230] Fasching L., Kapopoulou A., Sachdeva R., Petri R., Jonsson M.E., Manne C., Turelli P., Jern P., Cammas F., Trono D. (2015). TRIM28 represses transcription of endogenous retroviruses in neural progenitor cells. Cell Rep..

[231] Gaudi S., Guffanti G., Fallon J., Macciardi F. (2016). Epigenetic mechanisms and associated brain circuits in the regulation of positive emotions: A role for transposable elements. J. Comp. Neurol..

[232] Emera D., Yin J., Reilly S.K., Gockley J., Noonan J.P. (2016). Origin and evolution of developmental enhancers in the mammalian neocortex. Proc. Natl. Acad. Sci. USA.

[233] Notwell J.H., Chung T., Heavner W., Bejerano G. (2015). A family of transposable elements co-opted into developmental enhancers in the mouse neocortex. Nat. Commun..

[234] Turelli P., Playfoot C., Grun D., Raclot C., Pontis J., Coudray A., Thorball C., Duc J., Pankevich E.V., Deplancke B. (2020). Primate-restricted KRAB zinc finger proteins and target retrotransposons control gene expression in human neurons. Sci. Adv..

[235] Wang T., Medynets M., Johnson K.R., Doucet-O’Hare T.T., DiSanza B., Li W., Xu Y., Bagnell A., Tyagi R., Sampson K. (2020). Regulation of stem cell function and neuronal differentiation by HERV-K via mTOR pathway. Proc. Natl. Acad. Sci. USA.

[236] Nataf S., Uriagereka J., Benitez-Burraco A. (2019). The Promoter Regions of Intellectual Disability-Associated Genes Are Uniquely Enriched in LTR Sequences of the MER41 Primate-Specific Endogenous Retrovirus: An Evolutionary Connection Between Immunity and Cognition. Front. Genet..

[237] Suntsova M., Gogvadze E.V., Salozhin S., Gaifullin N., Eroshkin F., Dmitriev S.E., Martynova N., Kulikov K., Malakhova G., Tukhbatova G. (2013). Human-specific endogenous retroviral insert serves as an enhancer for the schizophrenia-linked gene PRODH. Proc. Natl. Acad. Sci. USA.

[238] Niu A.L., Wang Y.Q., Zhang H., Liao C.H., Wang J.K., Zhang R., Che J., Su B. (2011). Rapid evolution and copy number variation of primate RHOXF2, an X-linked homeobox gene involved in male reproduction and possibly brain function. BMC Evol. Biol..

[239] Naville M., Warren I.A., Haftek-Terreau Z., Chalopin D., Brunet F., Levin P., Galiana D., Volff J.N. (2016). Not so bad after all: Retroviruses and long terminal repeat retrotransposons as a source of new genes in vertebrates. Clin. Microbiol. Infect..

[240] Edelstein L.C., Collins T. (2005). The SCAN domain family of zinc finger transcription factors. Gene.

[241] Kaneko-Ishino T., Ishino F. (2012). The role of genes domesticated from LTR retrotransposons and retroviruses in mammals. Front. Microbiol..

[242] Grandi N., Tramontano E. (2018). Human Endogenous Retroviruses Are Ancient Acquired Elements Still Shaping Innate Immune Responses. Front. Immunol..

[243] Pastuzyn E.D., Day C.E., Kearns R.B., Kyrke-Smith M., Taibi A.V., McCormick J., Yoder N., Belnap D.M., Erlendsson S., Morado D.R. (2018). The Neuronal Gene Arc Encodes a Repurposed Retrotransposon Gag Protein that Mediates Intercellular RNA Transfer. Cell.

[244] Cardona-Alberich A., Tourbez M., Pearce S.F., Sibley C.R. (2021). Elucidating the cellular dynamics of the brain with single-cell RNA sequencing. RNA Biol..

[245] Ostapcuk V., Mohn F., Carl S.H., Basters A., Hess D., Iesmantavicius V., Lampersberger L., Flemr M., Pandey A., Thoma N.H. (2018). Activity-dependent neuroprotective protein recruits HP1 and CHD4 to control lineage-specifying genes. Nature.

[246] Bundo M., Toyoshima M., Okada Y., Akamatsu W., Ueda J., Nemoto-Miyauchi T., Sunaga F., Toritsuka M., Ikawa D., Kakita A. (2014). Increased l1 retrotransposition in the neuronal genome in schizophrenia. Neuron.

